# Deciphering the *Cis*-Regulatory Elements for XYR1 and CRE1 Regulators in *Trichoderma reesei*

**DOI:** 10.1371/journal.pone.0099366

**Published:** 2014-06-18

**Authors:** Rafael Silva-Rocha, Lilian dos Santos Castro, Amanda Cristina Campos Antoniêto, María-Eugenia Guazzaroni, Gabriela Felix Persinoti, Roberto Nascimento Silva

**Affiliations:** 1 Department of Biochemistry and Immunology, FMRP - University of São Paulo, Ribeirao Preto, São Paulo, Brazil; 2 Department of Biochemistry, FFCLRP - University of São Paulo, Ribeirao Preto, São Paulo, Brazil; Johns Hopkins University, United States of America

## Abstract

In this work, we report the *in silico* identification of the *cis*-regulatory elements for XYR1 and CRE1 proteins in the filamentous fungus *Trichoderma reesei*, two regulators that play a central role in the expression of cellulase genes. Using four datasets of condition-dependent genes from RNA-seq and RT-qPCR experiments, we performed unsupervised motif discovery and found two short motifs resembling the proposed binding consensus for XYR1 and CRE1. Using these motifs, we analysed the presence and arrangement of putative *cis*-regulatory elements recognized by both regulators and found that shortly spaced sites were more associated with XYR1- and CRE1-dependent promoters than single, high-score sites. Furthermore, the approach used here allowed the identification of the previously reported XYR1-binding sites from *cel7a* and *xyn1* promoters, and we also mapped the potential target sequence for this regulator at the *cel6a* promoter that has been suggested but not identified previously. Additionally, seven other promoters (for *cel7b*, *cel61a*, *cel61b*, *cel3c*, *cel3d*, *xyn3* and *swo* genes) presented a putative XYR1-binding site, and strong sites for CRE1 were found at the *xyr1* and *cel7b* promoters. Using the *cis*-regulatory architectures nearly defined for XYR1 and CRE1, we performed genome-wide identification of potential targets for direct regulation by both proteins and important differences on their functional regulons were elucidated. Finally, we performed binding site mapping on the promoters of differentially expressed genes found in *T. reesei* mutant strains lacking *xyr1* or *cre1* and found that indirect regulation plays a key role on their signalling pathways. Taken together, the data provided here sheds new light on the mechanisms for signal integration mediated by XYR1 and CRE1 at cellulase promoters.

## Introduction

*Trichoderma reesei* is a filamentous fungus extremely relevant to biotechnology due to its remarkable capability to produce a wide number of cellulolytic enzymes [1], [2]. This mesophilic organism is endowed with a tremendous repertoire of hydrolytic enzymes related to the deconstruction of lignocellulosic biomass from plants that are of high importance for biotech processes such as paper industry or fuel production [3], [4], [5]. Due to its elevated biotechnological potential, *T. reesei* has been extensively studied in the past decades as a model of cellulases and hemicellulases producing organism [6], [7], [8], [9]. This organism is endowed with different classes of biomass-related hydrolytic enzymes (here, collectively referred as cellulases), and special attention has been placed on enzymes such as endoglucanases (Cel7b, Cel5a, Cel12a, Cel61a and Cel45a), sGH61 polysaccharide monoxygenase (PMOs, Cel61a and Cel61b), exoglucanases (Cel7a and Cel6a), β-glucosidases (Cel3a and Cel1a), endo-β-1,4-xylanases (XYN1 and XYN2) and β-xylosidase (BXL1). From this particular set of enzymes, Cel7a, Cel6a, Cel7b and Cel5a are the most abundantly produced under inducing conditions such as growth in the presence of cellulose or sophorose (a glucose disaccharide produced during cellulose degradation; [6]).

In order to allow the ration engineering of new strains of *T. reesei* with enhance enzyme production levels, a great interest has been to elucidate the molecular mechanisms operating at the transcriptional network that controls the expression of cellulase genes in response to the cognate environmental conditions [6], [10], [11], [12], [13], [14]. These efforts have allowed the identification of many regulatory proteins and signalling pathways that are responsible for the coordination of cellulase expression in this fungus [15], [16], [17], [18], [19], [20]. For some of the enzymes mentioned above, at least three mechanistic steps take place at the promoter regions: chromatin reorganization, de-repression and induction [2], [6]. Chromatin reorganization is related to the dynamic positioning of nucleosomes in response to environmental or physiologic signals [21]. In Eukaryotes, nucleosomes are important players in gene regulation since their binding to DNA segments is able to lock the chromatin in a blocked state, where transcriptional factors (TFs) cannot interact with the *cis*-regulatory elements located in the region occupied by the nucleosome [22], [23], [24]. In fact, this process has been shown to regulate the basal expression levels of cellulase promoters in *T. reesei* [25], [26]. As a second step, de-repression is related to the increase in the basal promoter expression level in response to the removal of a repressive signal [6], . In the case of cellulases, carbon catabolite repression (CCR) is mediated by alternative carbon source of easy degradation, such as glucose [10], [20], [27], [28]. In this sense, the modulation of the promoter activity during CCR has been postulated to occur mainly through changing of the chromatin state of the target promoter [25], [26]. Finally, the third mechanism involves the induction of high promoter activities in response to some signals (in the case of cellulases, the enzyme substrates) that is mediated by general and specific TFs [6], [13], [17], [29].

The investigation of the regulatory network for cellulase expression in *T. reesei* has allowed the identification of several TFs related to each of the mechanisms described above. For instance, the XYR1 (*xylanase regulator 1*) transcriptional factor is the main positive regulator of cellulase expression in *T. reesei* [11], [19], and homologues of this protein performs the same role in other cellulase producing organisms such as *Aspergillus niger* and *Neurospora crassa* [30], [31]. XYR1 is a zinc binuclear cluster protein that is able to bind to several cellulase promoters and is virtually essential for full expression of these genes during growth under inducing conditions [11], [19]. Moreover, XYR1 production is also regulated at the transcriptional level by the carbon catabolite repressor CRE1 protein [17] and is repressed by the specific transcriptional factor ACE1 [32], [33]. CRE1 is a Cys2His2 type transcriptional factor that is responsible to mediate glucose dependent CCR at several cellulase promoters [10], [20]. This regulator is an homologue of the CCR protein Mig1 from *Saccharomyces cerevisiae* [34] and affects chromatin organization at target promoters in response to glucose [25], [26]. In turn, ACE1 contains three Cys2His2-type zinc fingers and also regulate other cellulase genes such as Cel7a and XYN1 apparently through the interference with the binding of XYR1 at targets promoters [29], [32]. In addition to the above cited proteins, additional regulators required for the expression of cellulase genes are the HAP2/3/5 complex, which is necessary to generate an open chromatin structure that is essential for full promoter activation, [26], [29], [35], [36] and the zinc binuclear cluster protein ACE2, which is a specific cellulase activator that only occurs in *T. reesei* [17], [36], [37].

Current available experimental data related to cellulase regulation in *T. reesei* clearly evidence sophisticate interplay between the characterized and unknown TFs at the target promoters to provide fine-tuning of enzyme production levels in this organism [6], [38]. Yet, the mechanisms by which the regulators at stake interact with the target *cis*-regulatory elements at each particular promoter are only beginning to be elucidated [11], [18], [36], [39], [40]. For instance, the consensus binding sequences of the two main cellulase regulators XYR1 and CRE1 (5′-GGCWWW-3′ and 5′-SYGGRG-3′, respectively) have been proposed on the basis of the comparison with homologous regulators form other organisms [41] or cannot be used to distinguish between genes regulated or not by these regulators [11]. Thus, quantitative information on the *cis*-regulatory elements associated with the interaction of XYR1 and CRE1 with their target promoters is crucial to improve engineering attempts to construct new cellulase responsive promoters [12], [42], [43] and to understand the role of these regulators in *T. reesei* at the global scale [10], [11]. In this work, we analysed four sets of co-regulated genes identified using RNA deep sequencing (RNA-seq) and Real Time quantitative PCR (RT-qPCR) from *T. reesei* cells grown in the presence of inducers (cellulose and sophorose) and a repressor (glucose) carbon sources. Separated analysis of the different datasets allowed the identification of new DNA motifs that were associated with the regulation by XYR1 and CRE1 in this organism. The nearly revealed motifs were used to generate, by the first time, Position Weight Matrixes (PWMs) representing the putative *cis*-regulatory elements recognized by both regulators. Furthermore, these PWMs were used to inspect the architecture of target promoters in co-expressed genes at the genomic scale, allowing the identification of the functional regulons of XYR1 and CRE1 in *T. reesei*. The PWMs generated here were able to successfully identify two previously characterized *cis*-regulatory elements at the *cel7a* and *xyn1* promoters [25], [29], as well as to find a new regulatory region at the *cel6a* promoter that agrees with previous *in vivo* data [26]. Taken together, the results provided here add valuable information on the regulatory scope of XYR1 and CRE1 in *T. reesei* at the genomic scape, revealing some important differences with other filamentous fungus [8], [30], [44] and providing new clues on the molecular mechanisms of promoter recognition by these regulators.

## Materials and Methods

### Experimental Datasets

**Figure 1:**
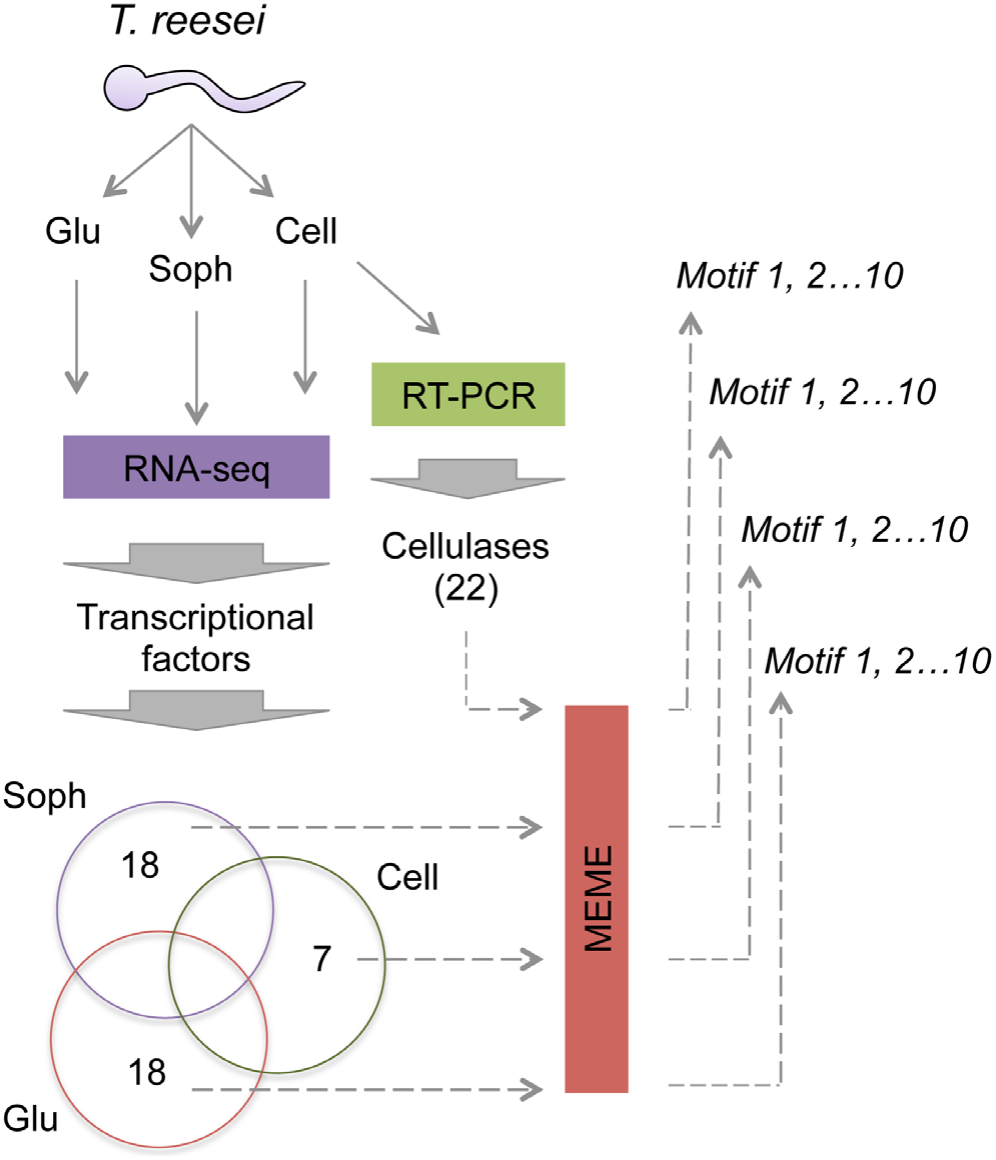
Schematic representation of the approach used for motif discovery in *T. reesei*. Three sets of co-regulated genes were retrieved from RNA-seq experiments of *T. reesei* cells grown on cellulose, sophorose or glucose [45]. Only up-regulated genes encoding for putative TFs were selected. A fourth set of genes are 22 cellulases analysed through RT-PCR experiments [46]. A 1.5 kb promoter region of each gene from the four groups was retrieved from the complete genome sequence [49] and used for motif discovery using MEME. From the resulting identified motifs, those sharing similarities with the reported binding consensus for XYR1 and CRE1 were selected for further analysis.

The analysis of *cis*-regulatory elements was performed using four groups of co-regulated genes identified using different experimental setups. Three groups represent genes encoding TFs that were identified using RNA-seq [45] from cells growing in cellulose (7 genes), sophorose (18 genes) or glucose (18 genes) as sole carbon sources and that were specifically up regulated. The lists of genes from these groups are given in **Tables S1 to S3** from Supporting Information. The fourth group is represented by 22 cellulases-encoding genes whose promoters are regulated by the XYR1 regulator [46]. For each of the 65 analysed genes, a 1.5 kb DNA sequence immediately upstream of ATG codons was retrieved from the complete genome sequence of *T. reesei* available at the JGI homepage (http://genome.jgi-psf.org/Trire2/Trire2.home.html) using *ad hoc Perl* scripts. These sequences are expected to contain the *cis*-regulatory elements for the different TFs acting on each gene. The four groups of fasta sequences where then used to identify conserved DNA motifs as described below. The overall approach used in this work is represented in **Fig. 1**.

### *De novo* Motif Discovery

**Figure 2:**
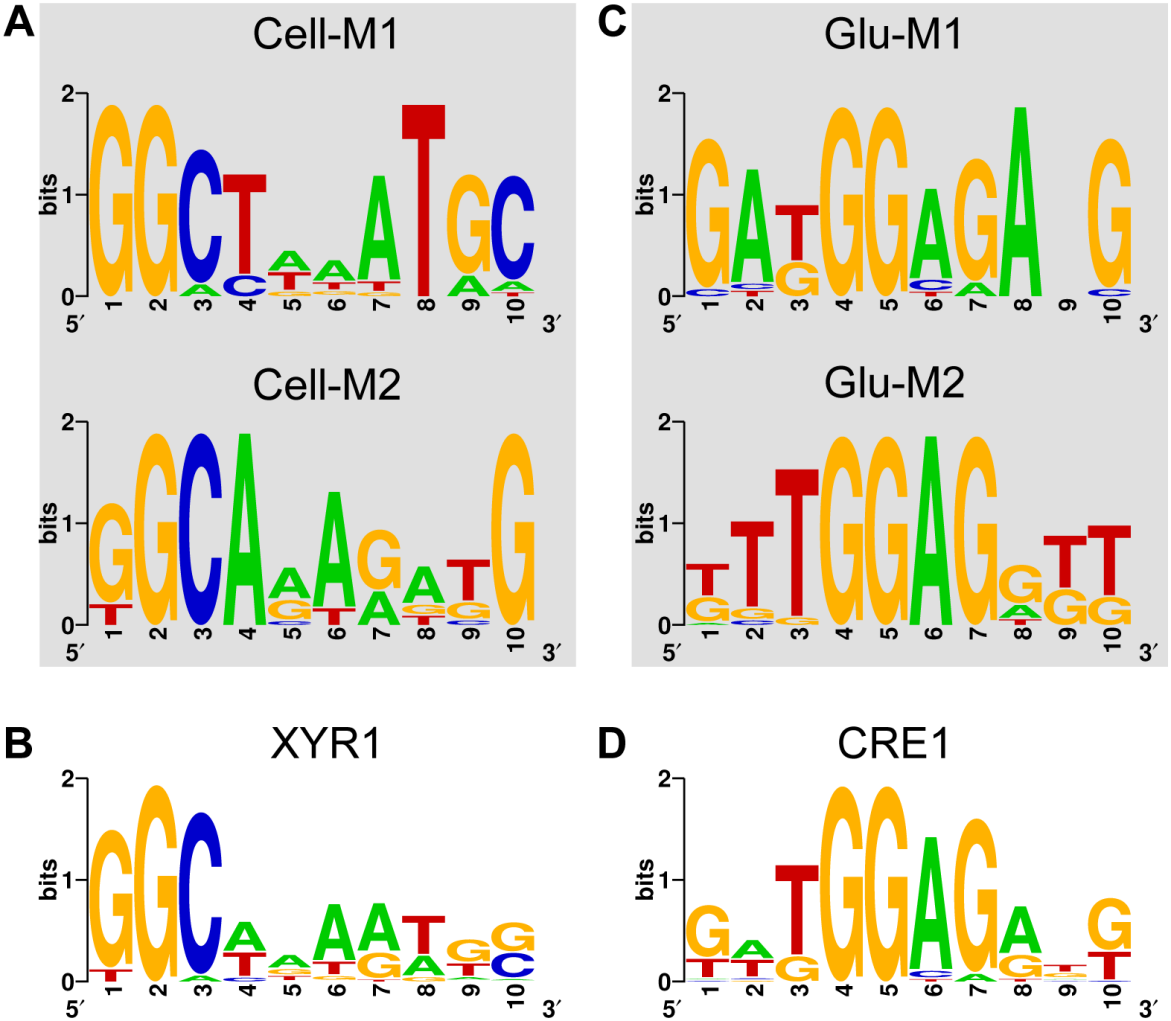
Motifs identified in the cellulose and glucose regulated genes. **A)** The two motifs identified in the promoter dataset of 22 cellulases that resemble the XYR1 consensus (5′-GGCWWW-3′) are shown. **B)** Combination of the Cell-M1 and Cell-M2 motifs to create the XYR1 consensus used to search for XYR1 binding sites in *T. reesei*. **C)** The two motifs identified using the promoters of TFs up regulated under glucose growth that share similarity to the proposed CRE1-binding consensus (5′-SYGGRG-3′) are shown. **D)** Representation of the consensus resulting from the combination of Glu-M1 and Glu-M2 motifs.

In order to identify new *cis*-regulatory elements in the four regulons assayed, we analysed the promoter sequences using the MEME tool [47]. For MEME analysis, we set the parameters to search for short DNA motifs (6 to 10 nt in length) expected to occur zero or one time per sequence at forward or reverse strand, allowing a maximal of ten different motifs to be reported by the program. From the resulting outputs, motifs displaying similarities with the DNA binding sites of XYR1 and CRE1 from *T. reesei* were selected. In cases where similar DNA consensus for the regulators appeared on different motif outputs, the aligned sequences were merged to create a single motif dataset. The resulting datasets were used to construct Position Weight Matrixes (PWM) by extracting the information content of the sequence alignments as described previously [48]. For XYR1, a PWM representing the first 8-nt of the motif (**Fig. 2B**) was used, while for CRE1, a PWM containing the complete 10-nt motif (**Fig. 2D**) was constructed. The two resulting PWMs (named PWM_XYR1_ for XYR1 and PWM_CRE1_ for CRE1 regulators) were used for further analysis.

### Genome-wide Analysis of *Cis*-regulatory Elements

The two PWMs generated using the motifs discovered with MEME were used to analyse the promoters of all annotated genes in the genome of *T. reesei* [49]. For this, promoters of 1.5 kb in length for the ∼9,000 genes of *T. reesei* were analysed to identify the best motif for both PWMs on each promoter. Next, the same dataset was re-analysed to identify multiple motifs per promoter with a score above a specific threshold, which was set to 6.2 for PWM_XYR1_ and 8.0 for PWM_CRE1_. The resulting identified sites were then analysed to identify adjacent *cis*-regulatory elements located within short distances (lower than 30 bp) with architectures similar to previously related functional sites for XYR1 and CRE1 regulators [11], [18], [25], [29], [37], [50], [51]. Additionally, the same workflow (i.e., identification of the best site, mapping of multiple sites and the search for adjacent elements) was applied to the promoters of the 18 genes from the Glucose dataset and the 22 cellulases promoters using both PWMs. Finally, two additional datasets, representing genes differentially expressed by growth on cellulose, sophorose and glucose in *Δxyr1* and *Δcre1* mutants of *T. reesei,* were inspected as described above to determine genes potentially and directly regulated by XYR1 (in the case of cellulose and sophorose growth conditions) and CRE1 (for glucose growth).

## Results and Discussion

### Discovery of Putative *Cis*-regulatory Elements in Co-regulated Genes in *T. reesei*

In order to gain quantitative information on the *cis*-regulatory elements of XYR1 and CRE1 in *T. reesei*, we used four different datasets of co-regulated genes to search for short repetitive DNA motifs potentially recognized by these regulators. For this, we used RNA-seq data from *T. reesei* cells grown on cellulose, sophorose and glucose as sole carbon sources [45]. Raw sequence data and count data for all samples are available at Gene Expression Omnibus (GEO database) under the accession number GSE53629. Within the differentially expressed genes identified in each condition, we selected only those encoding for TFs and that were up regulated in the different carbon sources. This procedure leads to the identification of 7, 18 and 18 TF-encoding genes on cellulose, sophorose and glucose growth conditions, respectively (**Table S1–S3**). It is worth mentioning that we focussed on TF-encoding genes since they could mediate the regulation by XYR1 or CRE1 in an indirect way [6]. In addition to these three datasets, we used a fourth group formed by 22 cellulase-encoding genes whose expression was impaired in a strain of *T. reesei* lacking a functional *xyr1* gene [46]. Using these datasets, we could then search for DNA motifs that are similar between the different group of genes and those which are specific to each experimental condition. The overall strategy used here is described schematically in **Fig. 1**. For motif discovery, we used MEME software [47] set to find short DNA segments (from 6 to 10 nt in length) that occurred zero or one time in each promoter. From the group of retrieved motifs (10 per dataset), we searched for those resembling the reported consensus for XYR1 (5′-GGCWWW-3′, where W stands for “A” or “T”) and CRE1 (5′-SYGGRG-3′, where refers to S is for “G” or “C”, Y for “C” or “T” and R for “A” or “G”) [11], [41]. In the case of the genes from RNA-seq experiments related to cells grown on cellulose or sophorose, we found no motif that resembled either consensus sequence. However, in the case of the 22 cellulases genes dependent of XYR1 [46] we were able to identify two motifs (named Cell-M1 and Cell-M2, **Fig. 2A**) showing a highly conserved GGC core followed by an AT rich region [11]. Each of these motifs was present in all the 22-cellulase promoters analysed, and they mainly diverged in the bases that were conserved at the 3′-end of the sequences (**Fig. 2A**). Then, the DNA sequences of the Cell-M1 and Cell-M2 motifs were merged to generate consensus that potentially represents the binding site for XYR1 in *T. reesei* (**Fig. 2B**).

On the other hand, when the short DNA motifs discovered by MEME on the Glucose dataset were analysed, we again could find two motifs (Glu-M1 and Glu-M2) that resembled the reported consensus sequence for CRE1 (**Fig. 2C**). These motifs have a core GGAG sequence at positions 4 to 7 that matches the expected GGRG consensus. However, although position 3 was expected to be occupied by a T or a C base, the most frequent base found at this location was a T, followed by a G, which was the second most represented base nucleotide (**Fig. 2C**). The region formed by these 5 bases (from 3 to 7) was the most conserved over the motifs, as shown by the consensus generated by the joint of their sequences (**Fig. 2D**). The position 2, which was expected to be occupied by a G or a C, was less conserved compared to the core region but displayed a slight preference for A and T bases (**Fig. 2D**). Finally, since these motifs were only detected on the Glucose promoter dataset and due to their high homology with the consensus for CRE1 binding site, we suggest that the motif in **Fig. 2D** in fact represents the *cis*-regulatory element recognized by this protein in *T. reesei*. Taken together, XYR1 and CRE1 motifs (**Fig. 2B and 2D**) display the first high-resolution representation of the binding sites recognized by XYR1 and CRE1, respectively, in *T. reesei,* and are valuable tools to investigate their regulons in this organism.

### Determining the Architecture of *Cis*-regulatory Elements for XYR1 and CRE1

After identifying the putative binding consensus of XYR1 and CRE1 in *T. reesei*, we decided to decipher how these regulators recognize their target promoters. While several works have tried to understand this process at the global scale, a unified model for protein-DNA interaction for these regulators is still not available [6], [38]. For instance, several reports support the notion that functional XYR1-binding sites have to be arranged in a specific way, such as inverted repeats [29], [52]. Alternatively, Furukawa and colleagues (2009) suggested that XYR1 regulated promoters are endowed with a higher number of single DNA sequences matching the GGCWWW consensus than the background genome. In the same way, promoter recognition by CRE1 has been explained in terms of single sites or repeated sites (either inverted or direct) spaced shortly from one another [10], [20], [25], [27], [39]. Since the two motifs generated here (**Fig. 2B and 2D**) represents a higher resolution description of the binding sites for both regulators, we resolved to investigate the role of single and multi-sites for the regulation of target promoters in their respective datasets. Following first the assumption that single sites would be enough to direct the regulators to their targets, one would expect that regulated promoters would harbour binding sites with higher affinity than un-regulated ones [53]. Using the score obtained from the specific Position Weight Matrixes (PWMs) representing XYR1 and CRE1 binding consensus as indicative of the relative TF-binding affinity [54], we accessed the score of identified sites at the genome scale and on the specific datasets (i.e., the group of 22 cellulases for XYR1 sites and the group of TFs up-regulated under Glucose growth for CRE1). For this, we identified only the best hit per promoter according to the two PWMs.

**Figure 3:**
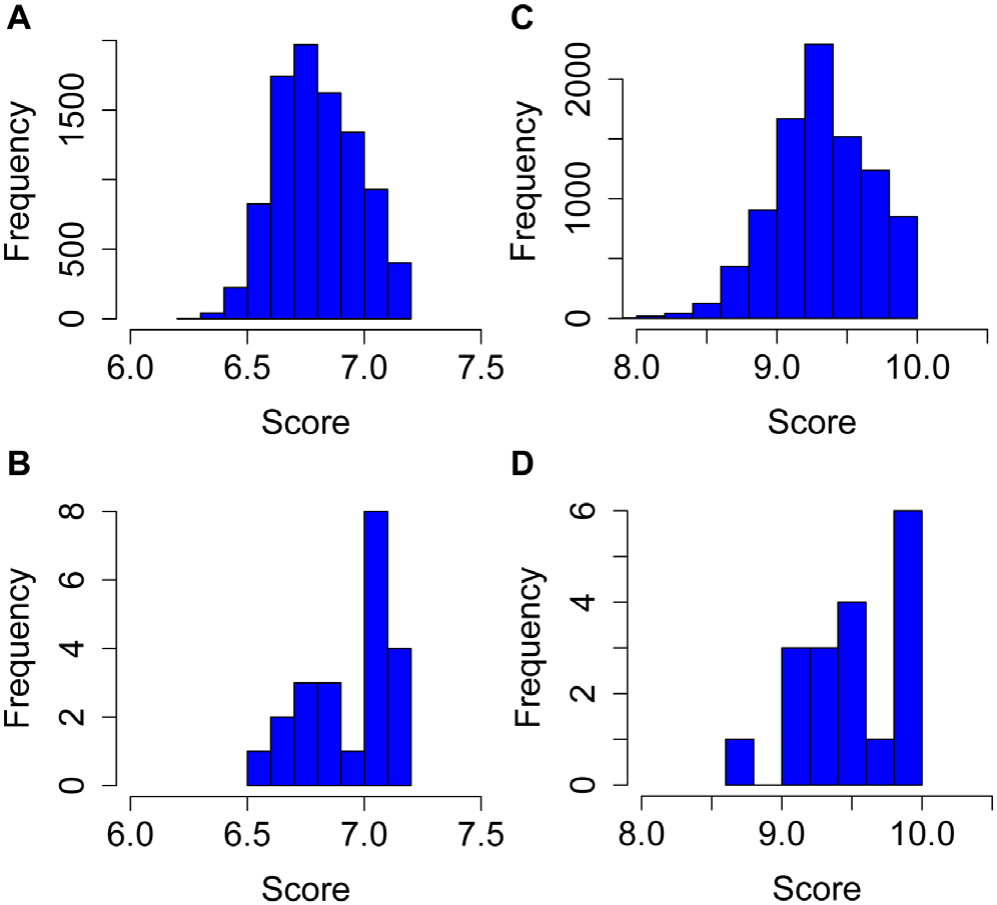
Search for single XYR1 and CRE1 binding sites on different promoter datasets. For the analysis, only the best site was retrieved for each studied promoter. **A**) Distribution of XYR1-binding sites score for all genes from the *T. reesei* genome. **B**) Score of XYR1-binding sites at the 22 cellulase promoters. **C**) Distribution of CRE1-bindind sites scores at the genome scale. **D**) Scores of CRE1-bindind sites found at the 18 promoters from the glucose dataset.

**Figure 4:**
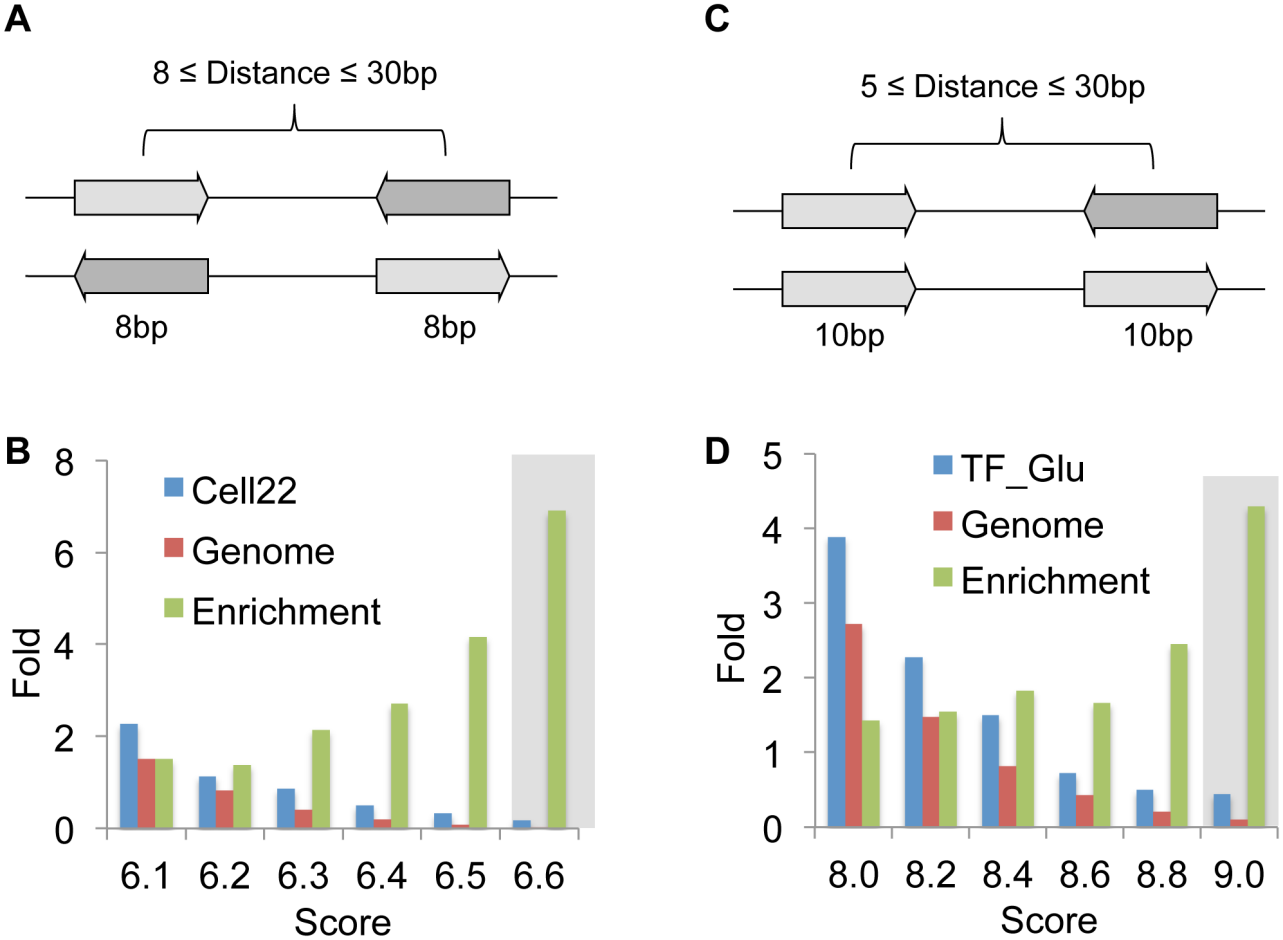
Search for repeated XYR1 and CRE1 binding sites on different datasets. **A**) In the XYR1, both inverted and everted sites were considered and only sites within a distance between 8 and 30 bp were taken. **B**) Representation of repeated binding sites at the cellulase promoters and at the genome scale. The y-axis (fold) represents the number of sites identified relative to the number of promoters from the datasets. The enrichment group represents the rate between sites per promoters from the cellulase promoters and the corresponding valued from the genome group. Grey shaded region highlight the score with higher enrichment. **C**) For the prediction of CRE1-binding sites, both inverted and direct repeats spaced between 5 to 30 bp were considered. **D**) Representation of sites per promoters and the enrichment at the glucose dataset vs. the genome, calculated as in **B**.

As shown in **Fig. 3A**, the putative binding sites for XYR1 at the genome scale followed a normal-like distribution with scores ranging from ∼6.2 to 7.3 and a peak near 6.7. However, when the promoter of the 22 cellulase genes were analysed, we found a much arrowed distribution with two peaks, one close to 6.7 and the other around 7 (**Fig. 3B**). In fact, this analysis reveals that nearly half of the cellulase promoters are endowed with a site with score above 7, while a considerably lower portion of the entire genome presents sites above this score. In the case of CRE1, the same pattern was observed, since a large portion of the Glucose promoter dataset displayed binding sites with scores between 9.8 and 10, while sites in this range were less frequent at the genome scale (**Fig. 3C–D**). In this way, these data strengthen the notion that single, high-affinity sites could be used as good descriptors of promoters targeted by XYR1 and CRE1 [11]. Yet, since the high score sites were still very abundant at the genomic level, we decided to investigate binding sites arranged in specific architectures proposed previously [6], [11], [18], [29], [55]. In the case of XYR1 binding sites, we focused on both inverted and everted repeats located within in a distance of between 8 to 30 nt from each other (**Fig. 4A**). We then searched for sites that fulfilled this requirement and used different thresholds in terms of score of both sites (ranging from 6.1 to 6.6). As shown in **Fig. 4B**, as expected, increasing threshold values generated the identification of fewer sites per promoters at both the cellulase group and at the genome scale. However, at higher thresholds we observed an enrichment of sites at the cellulase promoters in comparison to genes of *T. reesei* genome (**Fig. 4B**). In fact, one of the promoters that passed the 6.6 threshold value is from the Cel7a gene, which encodes one of the most abundant cellulase genes produced by *T. reesei* [6]. For the inspection of CRE1 binding sites, we searched for sites spaced within a distance of between 5 to 30 nt and both inverted and direct repeats were considered ([56]; **Fig. 4C**). As in the case of XYR1, the same tendency for enrichment of sites at the target promoters was observed, with an augmentation level of more than 4 when the score 9.0 was applied (**Fig. 4D**). These results show that searching for dual binding sites allowed a better definition of XYR1 and CRE1 targets. It is worth to notice that the disposition of the binding sites seemed to be more important than the PWM score itself, since thresholds near the average values of the genome distribution still provided a high enrichment in the target promoters (**Fig. 4B and 4D**). All together, the data provided here suggest that dual binding sites are more relevant for the recognition of the target promoters by XYR1 and CRE1 than single sites.

### Inspection of Cellulase Promoters for XYR1 and CRE1 Binding Sites

**Figure 5:**
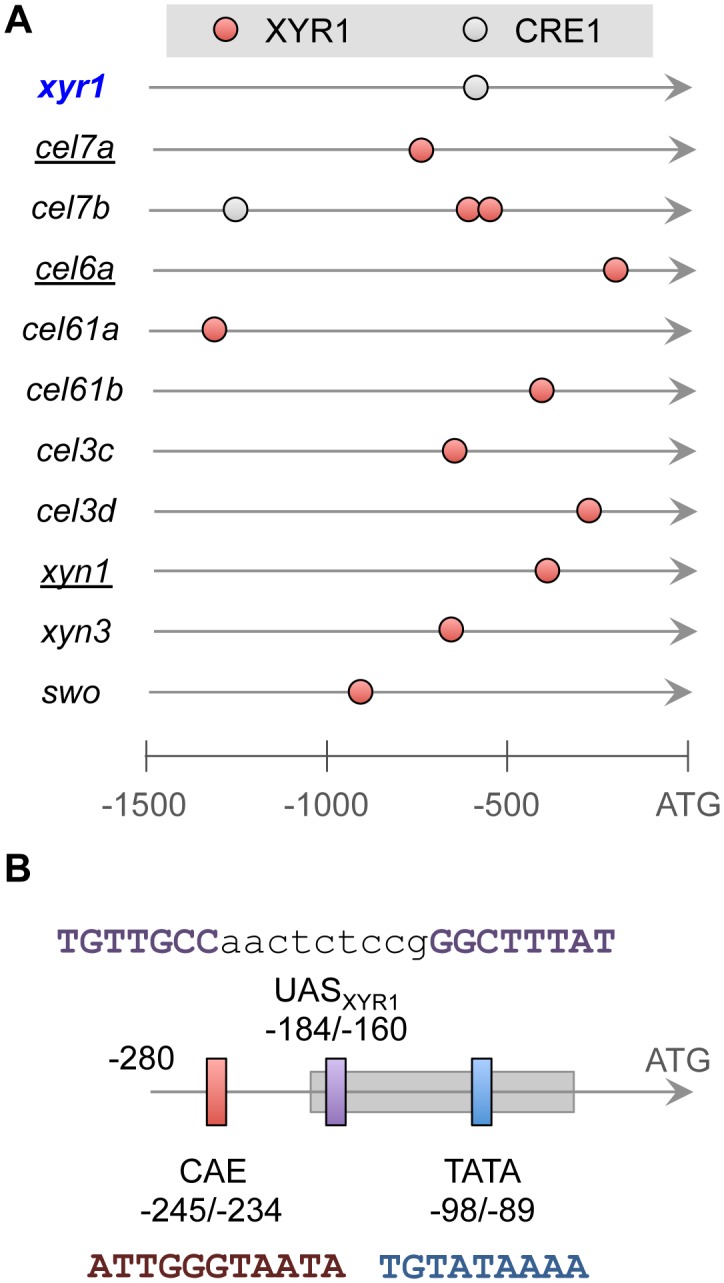
Identification of XYR1 and CRE1-binding sites at target promoters. **A**) Representation of the binding sites found at the promoters of 10 cellulase encoding-genes and at promoters of the *xyr1* gene. Each circle represent a binding site formed by repeats of the core sequences recognized by the two regulators. **B**) Zoom in at the promoter region of *cel6a* gene, showing the CAE (vertical red bar), the TATA-box (vertical blue bar), the nucleosome −1 binding region (horizontal grey rectangle) and the nearly identified binding site for XYR1, labelled as UAS_XYR1_. The DNA sequences of each regulatory element are shown [26]. Shown positions are relative to the start codon (ATG) of the *cel6a* gene.

Once we defined the relevant architecture of the *cis*-regulatory elements potentially recognized by XYR1 and CRE1, we decided to search for the presence of these elements in the promoters of the cellulase-encoding genes. As discussed before, it is well known that cellulase-encoding genes are controlled at the transcriptional level by induction in the presence of the substrates (such as cellulose and sophorose) and repression mediated by a preferred carbon source such as glucose [6], [38]. Whereas the participation of XYR1 and CRE1 regulators have been characterized for the induction and repression of cellulase promoters, respectively, remains an open question about which promoters are directly recognized by these proteins and which ones are regulated through indirect mechanisms involving yet unknown TFs [6], [38]. Up to now, *in vivo* and *in vitro* evidence for direct interaction between XYR1 and CRE1 have been reported for promoters such as *cel7a* [18], [19], *xyn1* [13], [19], [29] and proposed for *cel6a* [26], [40], [57]. In this way, we proceeded to the identification of potential XYR1 and CRE1 binding sites on 22 cellulase promoters using the architectures defined in the previous section, and we contrasted results with the information available in literature for the three characterized promoters. In addition, we inspected the promoter region of the *xyr1* gene, since CRE1 has been reported to affect its expression [17], [58]. As represented in **Fig. 5A**, using a threshold value of 6.4 for XYR1 binding sites, we were able to identity dual elements in 10 cellulase promoters. While most of the promoters presented a single dual site, the *cel7b* promoter displayed two sites that were shortly spaced and located about 600 bp upstream of the gene start codon (**Fig. 5A**). In addition, most of the identified promoters (9 out of 10) presented a putative XYR1 binding site located less than 1 kb from the ATG codon. Another interesting finding was that the promoters of three (Cel7a, Cel6a, Cel7b) of the four most efficient cellulolytic enzymes produced by *T. reesei* presented a putative dual XYR1 binding site as detected using the searching approach presented here.

Comparison of the binding sites found *in silico* with those previously characterized at some cellulase promoters showed a remarkable level of agreement. First, in the case of the *xyn1* promoter, a GGCTAA-box formed by two inverted repeats of the GGCWWW element and located around position −410 was shown to be required for XYR1 interaction *in vivo* and *in vitro* [29]. Using the searching methodology described here, the same sequence was identified as the putative XYR1 binding site (**Fig. 5A**). In the case of the *cel7a* promoter, two potential single binding sites for XYR1 have been proposed as functional at positions −320 and −733, but no direct evidence for their role was provided yet [25]. Our *in silico* analysis of the *cel7a* promoter revealed a high-score direct repeat site that includes at its 3′ region the −733 site previously reported. Finally, the case of the *cel6a* promoter represents a more interesting example. Previous analysis have revealed the existence of a region named CAE (for *cbh2* activating element) between positions −234 and −245 that is essential for the induction of this promoter in response to the presence of cellulose and sophorose [51], and this region was found to be regulated by the HAP2/3/5 complex and a yet uncharacterized protein [26]. By studying the nucleosome occupancy of the *cel6a* promoter, Zeilinger and colleagues (2003) found that the CAE is located in a nucleosome-free region and that proteins binding to this element controls the assemble of a nucleosome (named nucleosome −1) covering a region from −192 to −49 that includes the TATA-box [26]. These authors proposed an induction model where the removal of the nucleosome −1 is necessary to allow the interaction of proteins with TATA-box to allow the induction of the *cel6a* promoter in response to cellulose and sophorose. However, the target sequence of the XYR1 regulator at this promoter has not yet been identified, although the available data suggest that it would bind somewhere downstream the CAE region [26], [51]. The search for XYR1 binding sites used here allowed the identification of an everted repeat at the *cel6a* promoter at the position −160 to −184, which agrees perfectly with the current available information on the regulation of this promoter. It is worth to notice that everted binding sites are recognized by other zinc finger proteins [56], but XYR1 has not been associated with these elements so far. **Fig. 5B** summarizes the putative *cel6a* promoter architecture, including the nearly identified potential XYR1 binding site (named UAS_XYR1_) and the *cis*-regulatory elements characterized previously [26], [51].

When CRE1 binding sites were investigated in the cellulase dataset, only the *cel7a* promoter revealed an element that passed the stringent used criteria (**Fig. 5A**). However, most of the CRE1 binding sites proposed or demonstrated in literature have a poly-G at the “GGRG” part of the consensus sequence, while the PWM_CRE1_ identified here has a clear preference for the GGAG sequence (**Fig. 2D**). Yet, direct interaction between CRE1 and target promoters has been demonstrated for *cel7a* promoter but not for *cel6a* [26], clearly suggesting that CCR mediated by CRE1 on cellulase genes should be exerted through indirect mechanisms. In this sense, the clear candidate to be the mediator of CRE1 regulation is XYR1 itself, which has been reported as affected by CRE1 [17], [58]. In fact, *xyr1* was found to be the top one up-regulated gene under growth on glucose in a strain of *T. reesei* lacking a functional CRE1 protein (Antonieto *et al*. 2014, manuscript in preparation). Quite surprisingly, the searching for CRE1 binding sites at the *xyr1* promoter retrieved just a *cis*-regulatory element (**Fig. 5A**), contrasting the previous prediction of 10 single sites found using the degenerated consensus for this regulator [58]. Taken together, these analyses suggest that the binding sites identified here represent high confidence binding sites for XYR1 and CRE1 in *T. reesei*.

### Genome-wide Identification of Potential XYR1 and CRE1 Targets

**Figure 6:**
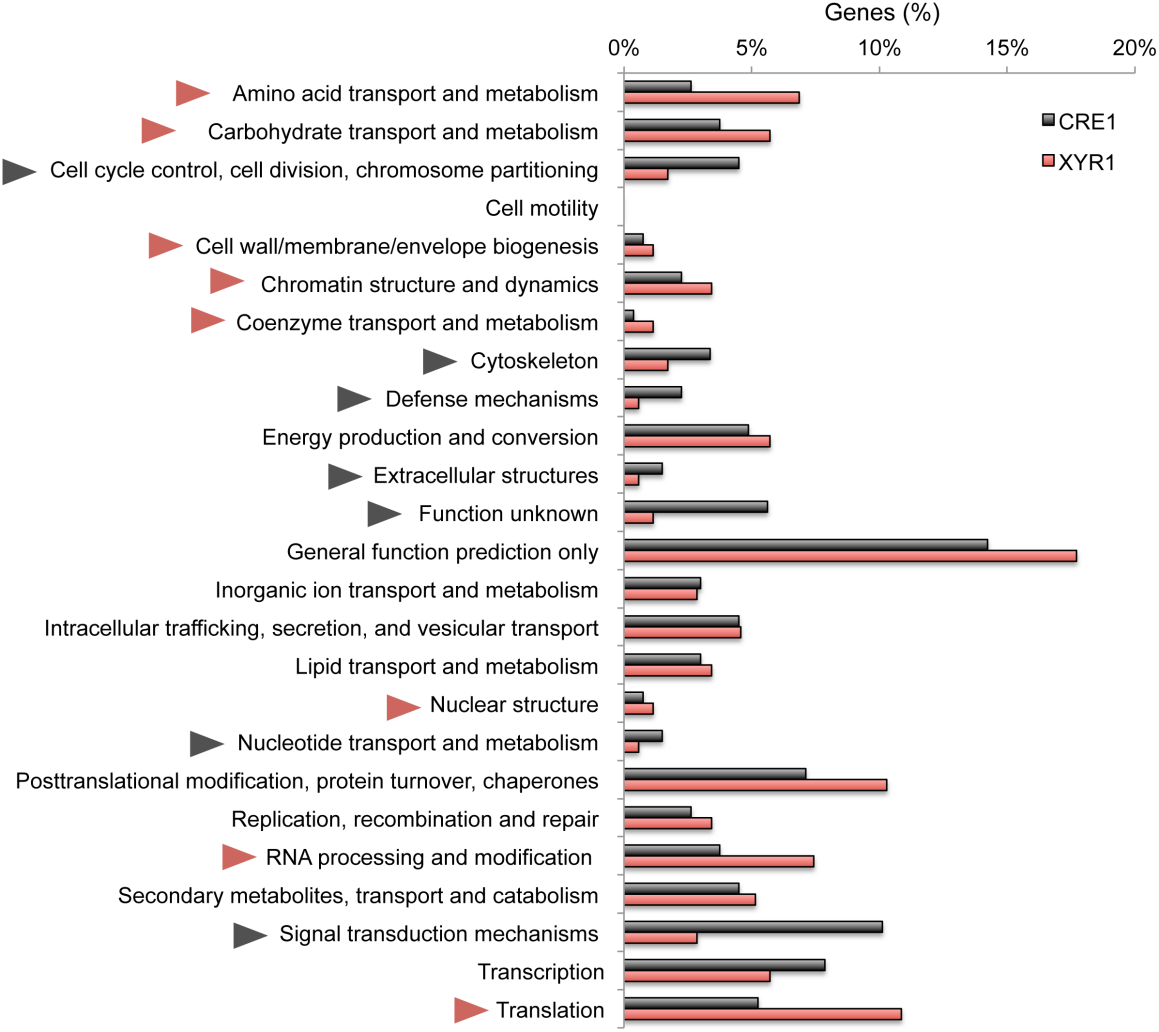
Defining the categories of genes potentially regulated directly by XYR1 and CRE1. Percentages of genes belonging to each functional category for both regulons are shown. Red triangles indicate functional categories enriched in the XYR1 regulon, while grey triangles point to those more abundant on the CRE1 regulon.

Once we defined the *cis*-regulatory architectures potentially recognized by XYR1 and CRE1, we performed a genome-wide inspection of potential target promoters for both regulators. For this, a list of 9,115 promoters relative to the annotated genes of *T. reesei* were analysed using the search criteria described in **Fig. 4**. Using a stringent threshold of 6.6 and 9.2 for XYR1 and CRE1 binding sites, respectively, we identified 233 genes potentially regulated by the former (**Table S4**) and 310 candidates for the latter (**Table S5**). Next, we classified the identified genes according to their functional categories (KOG), and then we compared the regulons to identify the difference in the functional scope of both regulators. As shown in **Fig. 6**, the potential XYR1 regulon is enriched mainly with genes related to the metabolization of carbohydrates and amino acids, chromatin structure and dynamics, RNA processing and modification and translation, among others. On the other hand, the putative CRE1 regulon showed a strong augmentation for genes related to signal transduction mechanisms and genes with unknown functions, cytoskeleton, cell cycle control and signal transduction mechanisms. While this analysis provided some clues about the potential targets of XYR1 and CRE1, it should be notice that it does not provide a full description of their regulons since additional TFs could mediate indirect regulation at target promoters [6], [59]. In this way, a better understanding of the functional scope of these proteins requires the integration of *in vivo* expression data, as is described below.

### Defining the Direct Role of XYR1 and CRE1 under Different Growth Conditions

In order to get an insight into the functioning of the regulon of XYR1 and CRE1 in *T. reesei,* we carried out a search for binding sites of these proteins in 6 sets of genes differentially regulated under different growth conditions. For this, we searched for XYR1 sites in the promoters of genes up and down regulated under growth in the presence of cellulose and sophorose in a strain of *T. reesei* lacking the functional *xyr1* gene, as determined through RNA-seq experiments (Castro *et al*., manuscript in preparation). In the same way, we surveyed CRE1 sites in promoters of genes up and down regulated during growth on glucose in a strain lacking the *cre1* gene (Antonieto *et al*., manuscript in preparation). Using these datasets, we found that between 13.6 and 15.8% of the genes differentially regulated in the wild type and *xyr1 *minus strain presented a putative XYR1 binding site. In the case of the *cre1* mutant experiments, between 8.9 and 13.9% of the promoters were endowed with a putative binding site for CRE1. The list of genes identified using this analysis along with their expression values determined using RNA-seq is provided in the Supporting Information (**Tables S6-S11**). Taken together, these data strongly indicated that indirect regulation plays an important role on control of target genes by XYR1 and CRE1 proteins in *T. reesei*.

### Conclusions

The data provide here addressed for the first time the quantitative identification of binding sites for XYR1 and CRE1 proteins, two general regulators that coordinate the expression of cellulase-encoding genes in *T. reesei* [10], [11], [58]. The main advantage of the approach used here was the utilization of sets of co-regulated genes to allow the unsupervised discovery of DNA motifs potentially related to the binding of TFs acting at the target group of genes. This analysis allowed us to define PWMs for XYR1 and CRE1 that are specific to *T. reesei*, eluding the bias generated by using consensus sequences determined in other organisms [41]. With these tools on hand, we could observe that while single sites worked generally well as descriptors of XYR1- and CRE1-regulated genes, repeated motifs shortly spaced and with different arrangements seemed to be more associated with promoters targeted by these regulators [42]. So, why some promoters would be endowed with high-score single sites while others presented repeated sites? In the face of the results presented here and those from the general model for cellulase induction currently available [6], [38], we propose a mechanistic model for XYR1 binding that could explain the presence of single or dual sites on cellulase promoters. The current proposed induction mechanism suggests that under a repression condition (i.e., in the presence of glucose) the production of cellulases is completely blocked, while under starvation conditions basal levels of these enzymes (mainly Cel7a and Cel6a) are produced. Subsequently, when the fungus finds cellulose, the produced enzymes act on this insoluble substrate to generate soluble inducers such as sophorose, which in turn would trigger the signal for high level of cellulase production [60], [61]. In our model, since we observed high-confidence dual binding sites on the *cel7a* and *cel6a* promoters as well as a strong CRE1 site in the *xyr1* regulatory region (**Fig. 5A**), starvation conditions would increase the levels of the XYR1 protein through the release of CCR mediated by CRE1 on its promoter. Next, increasing XYR1 levels would allow the formation of homodimers that would preferentially activate promoters endowed with *cis*-regulatory elements arranged as repeats, such as those for Cel7a and Cel6a. This would account for an increase in the basal expression of these proteins [60], [61]. Therefore, when these enzymes convert cellulose into the inducers such as sophorose, additional TFs able to sense this molecule would act in synergy with XYR1, perhaps through the formation of heterodimers, to active cellulase promoters formed by single of repeated sites, allowing thus the production of high levels of cellulases [6], [13]. Candidates for such promoter specific regulation include the nearly characterized BglR that regulates some β-glucosidase genes [16]. Evidently, new experimental approaches are required to get further insights into the mechanisms of signal integration present in the cellulase promoters in *T. reesei*, and we believe that the work reported here will contribute significantly for this task.

## Supporting Information

Tables S1 **Dataset of TFs up regulated in cellulose growth condition.**
(PDF)

Tables S2 **Dataset of TFs up regulated in sophorose growth condition.**
(PDF)

Tables S3 **Dataset of TFs up regulated in glucose growth condition.**
(PDF)

Tables S4 **Genome-wide prediction of XYR1 binding sites** ***in T. reesei*** **.**
(PDF)

Tables S5 **Genome-wide prediction of CRE1 binding sites** ***in T. reesei.***
(PDF)

Tables S6 **Prediction of XYR1 binding sites on genes down regulated in a** ***Δxyr1*** **mutant induced with** **cellulose.**
(PDF)

Tables S7 **Prediction of XYR1 binding sites on genes up regulated in a** ***Δxyr1*** **mutant induced with** **cellulose.**
(PDF)

Tables S8 **Prediction of XYR1 binding sites on genes down regulated in a** ***Δxyr1*** **mutant induced with** **sophorose.**
(PDF)

Tables S9 **Prediction of XYR1 binding sites on genes up regulated in a** ***Δxyr1*** **mutant induced with sophorose.**
(PDF)

Tables S10 **Prediction of CRE1 binding sites on genes down regulated in a** ***Δcre1*** **mutant induced with** **sophorose.**
(PDF)

Tables S11 **Prediction of CRE1 binding sites on genes up regulated in a** ***Δcre1*** **mutant induced with sophorose.**
(PDF)
