# Supplementary material for: Deciphering the Cis-Regulatory Elements for XYR1 and CRE1 Regulators in Trichoderma reesei
Source: PLoS One. 2014 Jun 18;9(6):e99366. doi: 10.1371/journal.pone.0099366 (PMC4062390; doi:10.1371/journal.pone.0099366)
Supplement: Tables S1 — Dataset of TFs up regulated in cellulose growth condition. (PDF) [file pone.0099366.s001.pdf]

Tables S1. Dataset of TFs up regulated in cellulose growth condition.

Protein ID	Description
**110152**	BZIP transcriptional regulator
**120698**	C2H2 transcriptional regulator
**108775**	Transcription factor AbaA
**68254**	Zn2Cys6 transcriptional regulator
**69972**	Zn2Cys6 transcriptional regulator
**121164**	Zn2Cys6 transcriptional regulator
**105269**	Zn2Cys6 transcriptional regulator
