# Supplementary material for: Deciphering the Cis-Regulatory Elements for XYR1 and CRE1 Regulators in Trichoderma reesei
Source: PLoS One. 2014 Jun 18;9(6):e99366. doi: 10.1371/journal.pone.0099366 (PMC4062390; doi:10.1371/journal.pone.0099366)
Supplement: Tables S2 — Dataset of TFs up regulated in sophorose growth condition. (PDF) [file pone.0099366.s002.pdf]

Tables S2. Dataset of TFs up regulated in sophorose growth condition.

Protein ID	Description
**73654**	BZIP transcriptional regulator
**67418**	C2H2 transcription factor
**120224**	C2H2 transcriptional regulator
**120428**	C2H2 transcriptional regulator
**120908**	myb transcriptional regulator
**80200**	transcription factor (Snd1/p100)
**62244**	Zn2Cys6 transcriptional regulator
**65746**	Zn2Cys6 transcriptional regulator
**66828**	Zn2Cys6 transcriptional regulator
**68455**	Zn2Cys6 transcriptional regulator
**55274**	Zn2Cys6 transcriptional regulator
**70351**	Zn2Cys6 transcriptional regulator
**58389**	Zn2Cys6 transcriptional regulator
**21997**	Zn2Cys6 transcriptional regulator
**123881**	Zn2Cys6 transcriptional regulator
**121107**	Zn2Cys6 transcriptional regulator
**73792**	Zn2Cys6 transcriptional regulator
**72611**	Zn2Cys6 transcriptional regulator
