# Supplementary material for: Deciphering the Cis-Regulatory Elements for XYR1 and CRE1 Regulators in Trichoderma reesei
Source: PLoS One. 2014 Jun 18;9(6):e99366. doi: 10.1371/journal.pone.0099366 (PMC4062390; doi:10.1371/journal.pone.0099366)
Supplement: Tables S3 — Dataset of TFs up regulated in glucose growth condition. (PDF) [file pone.0099366.s003.pdf]

Tables S3. Dataset of TFs up regulated in glucose growth condition.

Protein ID	Description
**119759**	BZIP transcriptional regulator
**21270**	CAP20 virulence factor
**78049**	Elongation factor Tu (G)
**75472**	Transcriptional regulator. unknown
**74346**	Translation elongation factor precursor from Aspergillus fumigatus
**57676**	Translation initiation factor 3. subunit i (elf-3i)
**74252**	Translation initiation factor 6 (eIF6) by homologyToThe corresponding protein in other eukaryotes
**54437**	Zn2Cys6 transcriptional regulator
**66047**	Zn2Cys6 transcriptional regulator
**55759**	Zn2Cys6 transcriptional regulator
**57534**	Zn2Cys6 transcriptional regulator
**102497**	Zn2Cys6 transcriptional regulator
**102499**	Zn2Cys6 transcriptional regulator
**109394**	Zn2Cys6 transcriptional regulator
**104182**	Zn2Cys6 transcriptional regulator
**112202**	Zn2Cys6 transcriptional regulator
**112499**	Zn2Cys6 transcriptional regulator
**105520**	Zn2Cys6 transcriptional regulator
