# Supplementary material for: Deciphering the Cis-Regulatory Elements for XYR1 and CRE1 Regulators in Trichoderma reesei
Source: PLoS One. 2014 Jun 18;9(6):e99366. doi: 10.1371/journal.pone.0099366 (PMC4062390; doi:10.1371/journal.pone.0099366)
Supplement: Tables S4 — Genome-wide prediction of XYR1 binding sites in T. reesei . (PDF) [file pone.0099366.s004.pdf]

Tables S4. Genome-wide prediction of XYR1 binding sites *in T. reesei*.

Protein ID	Site 1*	Site 2	Distance	Description	Functional category
**53947**	-1259	-1248	11	SWI-SNF chromatin-remodeling complex protein	Chromatin structure and dynamics
**53302**	-144	-133	11	Glycolate oxidase	Energy production and conversion
**73689**	-985	-964	21	FOG: Zn-finger	General function prediction only
**53576**	-228	-220	8	CREB binding protein/P300 and related TAZ Zn-finger proteins	Transcription
**54450**	-634	-611	23	mRNA capping enzyme, guanylyltransferase (alpha) subunit	RNA processing and modification
**119800**	-1179	-1164	15	Cell division control protein/predicted DNA repair exonuclease	Replication, recombination and repair
**74258**	-1477	-1451	26	Phenylalanyl-tRNA synthetase beta subunit	Translation, ribosomal structure and biogenesis
**103113**	-1156	-1140	16	Predicted short chain-type dehydrogenase	General function prediction only
**53601**	-637	-622	15	Synaptobrevin/VAMP-like protein	Intracellular trafficking, secretion, and vesicular transport
**62362**	-1019	-989	30	Calcium transporting ATPase	Inorganic ion transport and metabolism
**108012**	-524	-511	13	Prolylcarboxypeptidase (angiotensinase C)	Posttranslational modification, protein turnover, chaperones / General function prediction only
**71559**	-672	-645	27	Translation initiation factor 5A (eIF-5A)	Translation, ribosomal structure and biogenesis
**78560**	-424	-416	8	Predicted GTPase	General function prediction only
**108087**	-1131	-1119	12	Nuclear receptor coregulator SMRT/SMRTER, contains Myb-like domains	Transcription
**48951**	-993	-978	15	Protein involved in vacuolar polyphosphate accumulation, contains SPX domain	Inorganic ion transport and metabolism
**78753**	-251	-223	28	Cullins	Cell cycle control, cell division, chromosome partitioning
**78939**	-911	-891	20	RNA helicase BRR2, DEAD-box superfamily	RNA processing and modification
**71689**	-142	-127	15	Serine O-acetyltransferase	Amino acid transport and metabolism
**4479**	-978	-970	8	Small nuclear ribonucleoprotein (snRNP) Sm core protein	RNA processing and modification
**49696**	-207	-196	11	Molecular chaperone (DnaJ superfamily)	Posttranslational modification, protein turnover, chaperones
**122657**	-1199	-1174	25	Flavohemoprotein b5+b5R	Energy production and conversion
**64752**	-102	-92	10	DNA-directed RNA polymerase, subunit RPB10	Transcription
**79690**	-225	-201	24	Electron transfer flavoprotein ubiquinone oxidoreductase	Energy production and conversion

**122778**	-1050	-1039	11	Aldo/keto reductase family proteins	General function prediction only
**109404**	-1113	-1102	11	Splicing coactivator SRm160/300, subunit SRm300	RNA processing and modification
**22830**	-1338	-1324	14	Glutathione S-transferase	Posttranslational modification, protein turnover, chaperones
**4843**	-193	-170	23	Carbon-nitrogen hydrolase	Amino acid transport and metabolism
**4843**	-170	-159	11	Carbon-nitrogen hydrolase	Amino acid transport and metabolism
**65921**	-226	-197	29	3-Methylcrotonyl-CoA carboxylase, biotin-containing subunit/Propionyl-CoA carboxylase, alpha chain/Acetyl-CoA carboxylase, biotin carboxylase subunit	Amino acid transport and metabolism / Lipid transport and metabolism
**65921**	-197	-177	20	3-Methylcrotonyl-CoA carboxylase, biotin-containing subunit/Propionyl-CoA carboxylase, alpha chain/Acetyl-CoA carboxylase, biotin carboxylase subunit	Amino acid transport and metabolism / Lipid transport and metabolism
**109793**	-1194	-1184	10	Uncharacterized conserved protein	Function unknown
**66256**	-328	-306	22	Dehydrogenases with different specificities (related to short-chain alcohol dehydrogenases)	Secondary metabolites biosynthesis, transport and catabolism
**66311**	-757	-732	25	Predicted transporter (major facilitator superfamily)	General function prediction only
**123124**	-1362	-1336	26	Serine/threonine protein kinase	General function prediction only
**66154**	-1119	-1099	20	Nucleolar protein-like/EBNA1-binding protein	RNA processing and modification
**55263**	-182	-165	17	Ubiquitin-conjugating enzyme-related protein Ft1, involved in programmed cell death	Posttranslational modification, protein turnover, chaperones
**55034**	-999	-988	11	Sexual differentiation process protein ISP4	Signal transduction mechanisms
**123468**	-1107	-1088	19	IMP dehydrogenase/GMP reductase	Nucleotide transport and metabolism
**51420**	-1087	-1074	13	Ribosomal RNA adenine dimethylase	RNA processing and modification
**123561**	-990	-977	13	Subtilisin-like proprotein convertase	Posttranslational modification, protein turnover, chaperones
**81271**	-240	-230	10	Sorbitol dehydrogenase	Secondary metabolites biosynthesis, transport and catabolism
**51558**	-898	-888	10	Ankyrin repeat protein	General function prediction only
**81450**	-237	-225	12	Mitochondrial import inner membrane translocase, subunit TIM44	Intracellular trafficking, secretion, and vesicular transport
**111426**	-355	-329	26	Splicing coactivator SRm160/300, subunit SRm300	RNA processing and modification
**123884**	-206	-177	29	Queuine-tRNA ribosyltransferase	RNA processing and modification
**123989**	-757	-742	15	Uncharacterized conserved protein (tumor-rejection antigen MAGE in humans)	Function unknown

**75139**	-607	-579	28	Predicted E3 ubiquitin ligase	Posttranslational modification, protein turnover, chaperones
**75204**	-136	-111	25	Predicted Zn-finger protein	General function prediction only
**75205**	-1453	-1428	25	Nucleotide excision repair complex XPC-HR23B, subunit XPC/DPB11	Replication, recombination and repair
**104180**	-1410	-1384	26	Ankyrin	Cell wall/membrane/envelope biogenesis
**104211**	-1358	-1342	16	FKBP-type peptidyl-prolyl cis-trans isomerase	Posttranslational modification, protein turnover, chaperones
**75428**	-1338	-1309	29	Spliceosomal protein snRNP-U1A/U2B	RNA processing and modification
**56363**	-153	-145	8	Transcriptional regulator	Transcription
**23367**	-777	-766	11	Glucose dehydrogenase/choline dehydrogenase/mandelonitrile lyase (GMC oxidoreductase family)	General function prediction only
**82123**	-1336	-1317	19	Predicted transcription factor DATF1, contains PHD and TFS2M domains	Transcription
**70025**	-681	-658	23	Zinc-binding oxidoreductase	Energy production and conversion / General function prediction only
**82321**	-980	-956	24	Aquaporin (major intrinsic protein family)	Carbohydrate transport and metabolism
**57274**	-660	-650	10	WD40 repeat protein	General function prediction only
**57600**	-511	-494	17	Global transcriptional regulator, cell division control protein	Amino acid transport and metabolism
**75985**	-934	-915	19	Mitochondrial associated endoribonuclease MAR1 (isochorismatase superfamily)	General function prediction only
**105190**	-700	-670	30	Nuclear pore complex, Nup214/CAN component	Nuclear structure / Intracellular trafficking, secretion, and vesicular transport
**70956**	-273	-243	30	Cytochrome P450 CYP3/CYP5/CYP6/CYP9 subfamilies	Secondary metabolites biosynthesis, transport and catabolism
**76329**	-622	-601	21	Ran-binding protein RANBP1 and related RanBD domain proteins	Intracellular trafficking, secretion, and vesicular transport
**58638**	-1432	-1420	12	RNA polymerase II transcription factor complex subunit	Transcription
**121019**	-542	-530	12	Succinate dehydrogenase, flavoprotein subunit	Energy production and conversion
**58405**	-200	-174	26	U2 snRNP splicing factor, small subunit, and related proteins	RNA processing and modification
**58405**	-174	-155	19	U2 snRNP splicing factor, small subunit, and related proteins	RNA processing and modification
**6108**	-161	-146	15	SAM-dependent methyltransferases	Lipid transport and metabolism / General function prediction only
**121133**	-803	-781	22	Aspartyl protease	Posttranslational modification, protein turnover, chaperones

**76887**	-1145	-1128	17	Aspartyl protease	Posttranslational modification, protein turnover, chaperones
**121262**	-217	-200	17	Apoptosis-related protein/predicted DNA-binding protein	Cell cycle control, cell division, chromosome partitioning
**77009**	-347	-323	24	GTP-binding protein DRG2 (ODN superfamily)	Signal transduction mechanisms
**77038**	-1349	-1333	16	Synaptobrevin/VAMP-like protein SEC22	Intracellular trafficking, secretion, and vesicular transport
**77099**	-506	-485	21	Predicted hydrolases or acyltransferases (alpha/beta hydrolase superfamily)	General function prediction only
**121656**	-57	-47	10	Nuclear pore complex, Nup98 component (sc Nup145/Nup100/Nup116)	Nuclear structure / Intracellular trafficking, secretion, and vesicular transport
**107082**	-800	-783	17	FOG: RRM domain	General function prediction only
**22271**	-886	-866	20	Zinc-binding oxidoreductase	Energy production and conversion / General function prediction only
**107360**	-739	-730	9	Prolyl 4-hydroxylase alpha subunit	Amino acid transport and metabolism
**62071**	-299	-278	21	Dosage compensation regulatory complex/histone acetyltransferase complex, subunit MSL-3/MRG15/EAF3, and related CHROMO domain-containing proteins	Transcription / Chromatin structure and dynamics
**121826**	-454	-434	20	ATP-citrate lyase	Energy production and conversion
**121890**	-57	-34	23	Mitochondrial processing peptidase, alpha subunit	Posttranslational modification, protein turnover, chaperones
**121903**	-74	-53	21	Mitochondrial Fe-S cluster biosynthesis protein ISA2 (contains a HesB-like domain)	Intracellular trafficking, secretion, and vesicular transport / Energy production and conversion
**107547**	-397	-368	29	Translation initiation factor 4F, ribosome/mRNA-bridging subunit (eIF-4G)	Translation, ribosomal structure and biogenesis
**73643**	-143	-114	29	beta-1,6-N-acetylglucosaminyltransferase, contains WSC domain	Posttranslational modification, protein turnover, chaperones / Carbohydrate transport and metabolism
**53721**	-906	-895	11	Predicted alpha-helical protein, potentially involved in replication/repair	Replication, recombination and repair
**74080**	-1242	-1212	30	Panthothenate synthetase	Coenzyme transport and metabolism
**53503**	-1034	-1009	25	Predicted transporter (major facilitator superfamily)	General function prediction only
**119871**	-1301	-1286	15	Ras-related small GTPase, Rho type	General function prediction only
**78496**	-1333	-1323	10	Flavin-containing monooxygenase	Secondary metabolites biosynthesis, transport and catabolism
**78555**	-1231	-1223	8	Predicted esterase of the alpha-beta hydrolase superfamily (Neuropathy target esterase), contains cAMP-binding domains	General function prediction only
**22678**	-523	-504	19	Iron/ascorbate family oxidoreductases	Secondary metabolites biosynthesis, transport and catabolism / General function prediction only

**64347**	-1072	-1052	20	Projectin/twitchin and related proteins	Cytoskeleton
**122656**	-1006	-981	25	Splicing coactivator SRm160/300, subunit SRm300	RNA processing and modification
**64420**	-1315	-1305	10	WD40 repeat protein	Secondary metabolites biosynthesis, transport and catabolism / General function prediction only
**64720**	-795	-770	25	1-Acyl dihydroxyacetone phosphate reductase and related dehydrogenases	Secondary metabolites biosynthesis, transport and catabolism / General function prediction only
**64959**	-1145	-1134	11	Predicted phosphatase	General function prediction only
**64971**	-612	-594	18	Amino acid transporters	Amino acid transport and metabolism
**109285**	-1015	-994	21	Serine/threonine protein kinase	Signal transduction mechanisms
**50077**	-988	-974	14	Amino acid transporters	Amino acid transport and metabolism
**80058**	-21	-9	12	Predicted transporter (major facilitator superfamily)	General function prediction only
**65950**	-1300	-1276	24	Triglyceride lipase-cholesterol esterase	Lipid transport and metabolism
**4990**	-1316	-1294	22	Dehydrogenases with different specificities (related to short-chain alcohol dehydrogenases)	Secondary metabolites biosynthesis, transport and catabolism
**66296**	-627	-601	26	Eukaryotic-type DNA primase, large subunit	Replication, recombination and repair
**5063**	-270	-241	29	SWI-SNF chromatin-remodeling complex protein	Chromatin structure and dynamics
**120008**	-1332	-1317	15	Glucose dehydrogenase/choline dehydrogenase/mandelonitrile lyase (GMC oxidoreductase family)	General function prediction only
**55060**	-443	-415	28	Kinesin (SMY1 subfamily)	Cytoskeleton
**44928**	-718	-707	11	5-formyltetrahydrofolate cyclo-ligase	Coenzyme transport and metabolism
**55088**	-1346	-1321	25	NADH:ubiquinone oxidoreductase, NDUFS4/18 kDa subunit	Energy production and conversion
**110813**	-178	-169	9	Amino acid transporters	Amino acid transport and metabolism
**67938**	-823	-799	24	Reductases with broad range of substrate specificities	General function prediction only
**68364**	-970	-960	10	NIMA (never in mitosis)-related G2-specific serine/threonine protein kinase	Cell cycle control, cell division, chromosome partitioning
**68636**	-1332	-1320	12	Mitochondrial/chloroplast ribosomal protein S11	Translation, ribosomal structure and biogenesis
**111194**	-894	-880	14	Predicted GTPase	General function prediction only
**111216**	-1352	-1332	20	Histone H3 (Lys9) methyltransferase SUV39H1/Clr4, required for transcriptional silencing	Transcription / Chromatin structure and dynamics
**111345**	-930	-902	28	beta-1,6-N-acetylglucosaminyltransferase, contains WSC domain	Posttranslational modification, protein turnover, chaperones / Carbohydrate transport and metabolism
**68821**	-633	-623	10	UDP-glucuronosyl and UDP-glucosyl transferase	Energy production and conversion / Carbohydrate

					transport and metabolism
**68813**	-1163	-1137	26	Permease of the major facilitator superfamily	Carbohydrate transport and metabolism
**82026**	-1077	-1049	28	Predicted methyltransferase	General function prediction only
**23346**	-417	-388	29	Beta-N-acetylhexosaminidase	Carbohydrate transport and metabolism
**75380**	-710	-685	25	Permease of the major facilitator superfamily	Carbohydrate transport and metabolism
**104495**	-1079	-1071	8	RNA helicase	RNA processing and modification
**104557**	-303	-280	23	Dehydrogenases with different specificities (related to short-chain alcohol dehydrogenases)	Secondary metabolites biosynthesis, transport and catabolism
**72685**	-418	-406	12	6-phosphogluconate dehydrogenase	Carbohydrate transport and metabolism
**124031**	-1160	-1141	19	20S proteasome, regulatory subunit alpha type PSMA4/PRE9	Posttranslational modification, protein turnover, chaperones
**82235**	-240	-228	12	Maltase glucoamylase and related hydrolases, glycosyl hydrolase family 31	Carbohydrate transport and metabolism
**5916**	-1073	-1063	10	Histone acetyltransferase (MYST family)	Chromatin structure and dynamics
**5916**	-1063	-1035	28	Histone acetyltransferase (MYST family)	Chromatin structure and dynamics
**82385**	-1182	-1156	26	Methionyl-tRNA synthetase	Translation, ribosomal structure and biogenesis
**70646**	-526	-504	22	Beta, beta-carotene 15,15'-dioxygenase and related enzymes	Secondary metabolites biosynthesis, transport and catabolism
**57322**	-165	-155	10	Aromatic amino acid aminotransferase and related proteins	Amino acid transport and metabolism
**104831**	-1064	-1037	27	Splicing coactivator SRm160/300, subunit SRm300	RNA processing and modification
**104831**	-91	-66	25	Splicing coactivator SRm160/300, subunit SRm300	RNA processing and modification
**120789**	-1047	-1030	17	Alpha tubulin	Cytoskeleton
**120803**	-114	-89	25	Predicted divalent cation transporter	Inorganic ion transport and metabolism
**120821**	-310	-297	13	von Willebrand factor and related coagulation proteins	Defense mechanisms / Extracellular structures
**57433**	-777	-760	17	Subtilisin kexin isozyme-1/site 1 protease, subtilase superfamily	Posttranslational modification, protein turnover, chaperones
**105189**	-1282	-1252	30	20S proteasome, regulatory subunit beta type PSMB7/PSMB10/PUP1	Posttranslational modification, protein turnover, chaperones
**112520**	-827	-800	27	beta-1,6-N-acetylglucosaminyltransferase, contains WSC domain	Posttranslational modification, protein turnover, chaperones / Carbohydrate transport and metabolism
**105216**	-792	-779	13	Ankyrin	Cell wall/membrane/envelope biogenesis
**59056**	-1263	-1255	8	Acyl-CoA thioesterase	Lipid transport and metabolism

**58851**	-1279	-1267	12	Phosphatidylserine-specific receptor PtdSerR, contains JmjC domain	Chromatin structure and dynamics 7 Signal transduction mechanisms
**76491**	-759	-733	26	Regulator of Rac1, required for phagocytosis and cell migration	Signal transduction mechanisms
**76491**	-733	-714	19	Regulator of Rac1, required for phagocytosis and cell migration	Signal transduction mechanisms
**6103**	-43	-21	22	Synaptic vesicle transporter SVOP and related transporters (major facilitator superfamily)	General function prediction only
**73110**	-759	-737	22	Diamine acetyltransferase	Amino acid transport and metabolism
**76710**	-538	-525	13	RNA polymerase III, large subunit	Transcription
**121230**	-170	-145	25	Squalene monooxygenase	Lipid transport and metabolism
**106148**	-478	-461	17	DNA-binding protein C1D involved in regulation of double-strand break repair	Replication, recombination and repair
**77007**	-1457	-1433	24	FOG: RRM domain	General function prediction only
**77364**	-1150	-1125	25	Ubiquitin-specific protease	Posttranslational modification, protein turnover, chaperones
**60951**	-686	-656	30	Putative Zn-finger protein	General function prediction only
**121664**	-203	-182	21	Glutamate decarboxylase and related proteins	Amino acid transport and metabolism
**77647**	-255	-242	13	Threonine aldolase	Amino acid transport and metabolism
**60768**	-1129	-1112	17	Predicted peptidyl-tRNA hydrolase	Translation, ribosomal structure and biogenesis
**22264**	-915	-895	20	Apoptosis antagonizing transcription factor/protein transport protein	Transcription / Intracellular trafficking, secretion, and vesicular transport
**121901**	-1161	-1140	21	Translation initiation factor 3, subunit c (eIF-3c)	Translation, ribosomal structure and biogenesis
**61750**	-105	-76	29	Sphingolipid hydroxylase	Lipid transport and metabolism
**27599**	-1152	-1122	30	Mitochondrial Fe2+ transporter MMT1 and related transporters (cation diffusion facilitator superfamily)	Inorganic ion transport and metabolism
**60402**	-143	-127	16	Mitochondrial GTPase	Translation, ribosomal structure and biogenesis
**35202**	-190	-167	23	Helicases	Replication, recombination and repair
**61948**	-1449	-1440	9	Protein disulfide isomerase (prolyl 4-hydroxylase beta subunit)	Posttranslational modification, protein turnover, chaperones
**40290**	-251	-222	29	Putative translation initiation inhibitor UK114/IBM1	Translation, ribosomal structure and biogenesis
**55595**	-838	-822	16	Ca2+/H+ antiporter VCX1 and related proteins	Inorganic ion transport and metabolism
**21396**	-83	-63	20	unknown protein	-
**62475**	-555	-528	27	unknown protein	-

**78499**	-1234	-1224	10	unknown protein	-
**122178**	-777	-761	16	unknown protein	-
**108251**	-321	-310	11	unknown protein	-
**79238**	-1149	-1129	20	unknown protein	-
**122576**	-841	-819	22	unknown protein	-
**64172**	-99	-79	20	unknown protein	-
**65948**	-1228	-1204	24	unknown protein	-
**80340**	-864	-844	20	unknown protein	-
**123282**	-72	-58	14	unknown protein	-
**74576**	-661	-648	13	unknown protein	-
**55561**	-1198	-1183	15	unknown protein	-
**45153**	-136	-111	25	unknown protein	-
**68291**	-1167	-1137	30	unknown protein	-
**68930**	-70	-45	25	unknown protein	-
**111514**	-582	-564	18	unknown protein	-
**72526**	-236	-227	9	unknown protein	-
**72526**	-227	-206	21	unknown protein	-
**82032**	-609	-580	29	unknown protein	-
**104075**	-321	-293	28	unknown protein	-
**104399**	-1491	-1476	15	unknown protein	-
**104584**	-831	-810	21	unknown protein	-
**82227**	-659	-643	16	unknown protein	-
**82286**	-246	-236	10	unknown protein	-
**82286**	-236	-208	28	unknown protein	-
**124119**	-888	-877	11	unknown protein	-
**124119**	-660	-651	9	unknown protein	-
**112392**	-397	-384	13	unknown protein	-
**57527**	-1037	-1024	13	unknown protein	-

**112603**	-56	-39	17	unknown protein	-
**58244**	-1335	-1327	8	unknown protein	-
**121107**	-1258	-1246	12	unknown protein	-
**121121**	-83	-56	27	unknown protein	-
**105849**	-371	-353	18	unknown protein	-
**76820**	-622	-596	26	unknown protein	-
**73231**	-325	-296	29	unknown protein	-
**60337**	-109	-100	9	unknown protein	-
**106755**	-521	-496	25	unknown protein	-
**74223**	-396	-410	14	unknown protein	-
**108011**	-1026	-1013	13	unknown protein	-
**122262**	-1265	-1244	21	unknown protein	-
**63952**	-1010	-999	11	unknown protein	-
**64011**	-726	-715	11	unknown protein	-
**109317**	-150	-126	24	unknown protein	-
**80181**	-290	-266	24	unknown protein	-
**109790**	-1042	-1013	29	unknown protein	-
**109790**	-1013	-993	20	unknown protein	-
**109846**	-871	-863	8	unknown protein	-
**110127**	-345	-322	23	unknown protein	-
**123261**	-939	-920	19	unknown protein	-
**123261**	-681	-663	18	unknown protein	-
**110600**	-172	-145	27	unknown protein	-
**67605**	-716	-690	26	unknown protein	-
**110731**	-999	-979	20	unknown protein	-
**111059**	-298	-284	14	unknown protein	-
**111245**	-151	-124	27	unknown protein	-
**111442**	-976	-961	15	unknown protein	-

**69222**	-215	-186	29	unknown protein	-
**111739**	-466	-437	29	unknown protein	-
**69493**	-820	-804	16	unknown protein	-
**45445**	-271	-256	15	unknown protein	-
**104380**	-640	-614	26	unknown protein	-
**112122**	-1208	-1188	20	unknown protein	-
**124175**	-1324	-1299	25	unknown protein	-
**104838**	-1067	-1050	17	unknown protein	-
**57730**	-1036	-1028	8	unknown protein	-
**112518**	-931	-921	10	unknown protein	-
**105454**	-391	-369	22	unknown protein	-
**121082**	-825	-804	21	unknown protein	-
**59244**	-1101	-1090	11	unknown protein	-
**3488**	-642	-618	24	unknown protein	-
**60445**	-956	-929	27	unknown protein	-
**121471**	-1149	-1134	15	unknown protein	-
**77481**	-1168	-1152	16	unknown protein	-
**77780**	-747	-727	20	unknown protein	-
**107490**	-1327	-1313	14	unknown protein	-
**33650**	-167	-141	26	unknown protein	-
**69557**	-1061	-1033	28	unknown protein	-
**63382**	-1133	-1125	8	unknown protein	-
**26163**	-1074	-1058	16	unknown protein	-

*Positions of Sites 1 and 2 are relative to the gene start codon.
