# Supplementary material for: Deciphering the Cis-Regulatory Elements for XYR1 and CRE1 Regulators in Trichoderma reesei
Source: PLoS One. 2014 Jun 18;9(6):e99366. doi: 10.1371/journal.pone.0099366 (PMC4062390; doi:10.1371/journal.pone.0099366)
Supplement: Tables S5 — Genome-wide prediction of CRE1 binding sites in T. reesei. (PDF) [file pone.0099366.s005.pdf]

Tables S5. Genome-wide prediction of CRE1 binding sites *in T. reesei*.

Protein ID	Site 1*	Site 2	Distance	Description	Functional category
**54283**	-709	-700	9	Predicted carbohydrate kinase, contains PfkB domain	General function prediction only
**102572**	-335	-315	20	Parvulin-like peptidyl-prolyl cis-trans isomerase	Posttranslational modification, protein turnover, chaperones
**43817**	-912	-903	9	PHD Zn-finger protein	General function prediction only
**53576**	-872	-862	10	CREB binding protein/P300 and related TAZ Zn-finger proteins	Transcription
**102743**	-500	-489	11	von Willebrand factor and related coagulation proteins	Defense mechanisms / Extracellular structures
**102743**	-191	-183	8	von Willebrand factor and related coagulation proteins	Defense mechanisms / Extracellular structures
**44330**	-1165	-1155	10	Dual-specificity tyrosine-phosphorylation regulated kinase	General function prediction only
**74258**	-1111	-1084	27	Phenylalanyl-tRNA synthetase beta subunit	Translation, ribosomal structure and biogenesis
**2087**	-1354	-1345	9	Exosomal 3'-5' exoribonuclease complex, subunit Rrp43	Translation, ribosomal structure and biogenesis
**74486**	-247	-238	9	Uncharacterized conserved protein	Function unknown
**78176**	-1489	-1477	12	1,3-beta-glucan synthase/callose synthase catalytic subunit	Cell wall/membrane/envelope biogenesis
**107779**	-835	-829	6	Predicted alpha/beta hydrolase BEM46	General function prediction only
**122103**	-61	-55	6	Predicted nucleotidyltransferase	General function prediction only
**22538**	-1477	-1466	11	Vacuolar assembly/sorting protein PEP5/VPS11	Intracellular trafficking, secretion, and vesicular transport
**78817**	-56	-27	29	26S proteasome regulatory complex, ATPase RPT6	Posttranslational modification, protein turnover, chaperones
**79124**	-807	-798	9	Kinesin-like protein	Cytoskeleton
**122476**	-536	-530	6	Protein involved in autophagy and nutrient starvation	Posttranslational modification, protein turnover, chaperones
**22647**	-85	-58	27	Microfibrillar-associated protein MFAP1	Cytoskeleton
**108697**	-512	-505	7	Calcium-responsive transcription coactivator	Transcription
**108706**	-68	-62	6	Vacuolar sorting protein/ubiquitin receptor VPS23	Intracellular trafficking, secretion, and vesicular transport / Posttranslational modification, protein turnover, chaperones
**64111**	-423	-412	11	Structural maintenance of chromosome protein SMC5/Spr18, SMC superfamily	Replication, recombination and repair / Chromatin structure and dynamics / Cell cycle control, cell division, chromosome partitioning
**108784**	-657	-638	19	Zinc-binding oxidoreductase	Energy production and conversion / General function prediction only
**64193**	-1329	-1323	6	Transferrin receptor and related proteins containing the protease-associated (PA) domain	Posttranslational modification, protein turnover, chaperones / Inorganic ion transport and metabolism / General function prediction only

**64193**	-1323	-1317	6	Transferrin receptor and related proteins containing the protease-associated (PA) domain	Posttranslational modification, protein turnover, chaperones / Inorganic ion transport and metabolism / General function prediction only
**64193**	-1317	-1311	6	Transferrin receptor and related proteins containing the protease-associated (PA) domain	Posttranslational modification, protein turnover, chaperones / Inorganic ion transport and metabolism / General function prediction only
**22731**	-258	-252	6	Beclin-like protein	Signal transduction mechanisms
**4654**	-319	-306	13	Histidinol dehydrogenase	Amino acid transport and metabolism
**49832**	-839	-810	29	Translation initiation factor 2C (eIF-2C) and related proteins	Translation, ribosomal structure and biogenesis
**79673**	-677	-670	7	Serine/threonine protein kinase	Signal transduction mechanisms
**79741**	-266	-242	24	Sulfate/bicarbonate/oxalate exchanger SAT-1 and related transporters (SLC26 family)	Inorganic ion transport and metabolism
**122828**	-199	-186	13	Uncharacterized protein of the uridine kinase family	Nucleotide transport and metabolism
**79884**	-207	-193	14	Ubiquitin-like protein	Cell cycle control, cell division, chromosome partitioning / Posttranslational modification, protein turnover, chaperones
**80007**	-1059	-1040	19	WW domain binding protein WBP-2, contains GRAM domain	Signal transduction mechanisms
**4905**	-956	-947	9	VAMP-associated protein involved in inositol metabolism	Intracellular trafficking, secretion, and vesicular transport
**65969**	-224	-201	23	Cytoskeletal protein Adducin	Signal transduction mechanisms / Cytoskeleton
**4947**	-173	-167	6	TRAPP 20 K subunit	Intracellular trafficking, secretion, and vesicular transport
**27085**	-737	-707	30	KRR1-interacting protein involved in 40S ribosome biogenesis	Translation, ribosomal structure and biogenesis
**109779**	-134	-127	7	von Willebrand factor and related coagulation proteins	Defense mechanisms / Extracellular structures
**123030**	-796	-785	11	RhoGEF GTPase	Signal transduction mechanisms
**80231**	-133	-127	6	Hexokinase	Carbohydrate transport and metabolism
**66405**	-1363	-1355	8	Transcription initiation factor TFIID, subunit TAF5 (also component of histone acetyltransferase SAGA)	Transcription
**123114**	-1289	-1262	27	Molecular chaperone (HSP90 family)	Posttranslational modification, protein turnover, chaperones
**123226**	-754	-742	12	Neutral trehalase	Carbohydrate transport and metabolism
**120172**	-100	-92	8	Fe-S oxidoreductase	Energy production and conversion
**2384**	-543	-535	8	Signaling protein SWIFT and related BRCT domain proteins	Transcription / Replication, recombination and repair / Cell cycle control, cell division, chromosome partitioning / Signal transduction mechanisms
**55180**	-938	-921	17	COP9 signalosome, subunit CSN6	Signal transduction mechanisms / Posttranslational modification, protein turnover, chaperones
**55172**	-782	-767	15	Isoamyl acetate-hydrolyzing esterase and related enzymes	General function prediction only

**80833**	-1424	-1398	26	Chitinase	Carbohydrate transport and metabolism
**23050**	-1126	-1118	8	Small Nuclear ribonucleoprotein splicing factor	RNA processing and modification
**81102**	-1088	-1070	18	Flavin-containing monooxygenase	Secondary metabolites biosynthesis, transport and catabolism
**68107**	-979	-973	6	Ubiquitin-like/40S ribosomal S30 protein fusion	Translation, ribosomal structure and biogenesis / Posttranslational modification, protein turnover, chaperones
**81303**	-129	-108	21	Fumarate reductase, flavoprotein subunit	Energy production and conversion
**111102**	-308	-302	6	Glycosylphosphatidylinositol-specific phospholipase C	Signal transduction mechanisms
**111102**	-302	-296	6	Glycosylphosphatidylinositol-specific phospholipase C	Signal transduction mechanisms
**68622**	-1055	-1046	9	Uncharacterized conserved protein	Function unknown
**123705**	-1173	-1145	28	UDP-glucose/GDP-mannose dehydrogenase	Signal transduction mechanisms / Carbohydrate transport and metabolism
**111417**	-1083	-1077	6	Dehydrogenases with different specificities (related to short-chain alcohol dehydrogenases)	Secondary metabolites biosynthesis, transport and catabolism
**5656**	-178	-165	13	Predicted transporter (major facilitator superfamily)	General function prediction only
**69122**	-859	-848	11	Fatty acid desaturase	Lipid transport and metabolism
**69068**	-760	-751	9	Predicted ubiquitin regulatory protein	Posttranslational modification, protein turnover, chaperones
**81843**	-287	-275	12	Alkaline phosphatase	Inorganic ion transport and metabolism
**81859**	-559	-534	25	Ubiquitin carboxyl-terminal hydrolase	Posttranslational modification, protein turnover, chaperones
**123946**	-580	-562	18	Predicted dehydrogenase	Secondary metabolites biosynthesis, transport and catabolism
**104180**	-197	-173	24	Ankyrin	Cell wall/membrane/envelope biogenesis
**56792**	-747	-737	10	Predicted mitochondrial cholesterol transporter	Signal transduction mechanisms / Lipid transport and metabolism
**56515**	-1465	-1438	27	Mismatch repair ATPase MSH4 (MutS family)	Replication, recombination and repair
**120391**	-76	-61	15	Serine/threonine protein kinase	Signal transduction mechanisms
**57010**	-692	-686	6	Protein geranylgeranyltransferase Type I, beta subunit	Posttranslational modification, protein turnover, chaperones
**75397**	-428	-405	23	Vacuolar assembly/sorting protein VPS9	Intracellular trafficking, secretion, and vesicular transport
**2648**	-182	-160	22	Predicted component of NuA3 histone acetyltransferase complex	Chromatin structure and dynamics
**75444**	-89	-74	15	COP9 signalosome, subunit CSN4	Signal transduction mechanisms / Posttranslational modification, protein turnover, chaperones
**75444**	-42	-36	6	COP9 signalosome, subunit CSN4	Signal transduction mechanisms / Posttranslational modification, protein turnover, chaperones
**72661**	-106	-99	7	Serine/threonine protein kinase	Signal transduction mechanisms

**104535**	-1485	-1463	22	Telomerase elongation inhibitor/RNA maturation protein PINX1	RNA processing and modification / Cell cycle control, cell division, chromosome partitioning
**75568**	-1479	-1463	16	Thioredoxin-like protein	Posttranslational modification, protein turnover, chaperones
**2745**	-128	-119	9	Fumarase	Energy production and conversion
**124094**	-400	-394	6	Predicted methyltransferase	General function prediction only
**5927**	-259	-232	27	Serine carboxypeptidases (lysosomal cathepsin A)	Posttranslational modification, protein turnover, chaperones / Amino acid transport and metabolism
**70548**	-158	-152	6	Ras1 guanine nucleotide exchange factor	Signal transduction mechanisms
**124181**	-1059	-1044	15	Members of tubulin/FtsZ family	Cytoskeleton
**82499**	-201	-177	24	Predicted integral membrane protein	General function prediction only
**120623**	-901	-894	7	Nuclear receptor coregulator SMRT/SMRTER, contains Myb-like domains	Transcription
**120623**	-894	-887	7	Nuclear receptor coregulator SMRT/SMRTER, contains Myb-like domains	Transcription
**21752**	-1103	-1092	11	Amphiphysin	Intracellular trafficking, secretion, and vesicular transport
**46247**	-962	-939	23	WD40 repeat-containing protein	General function prediction only
**76117**	-159	-146	13	Defense-related protein containing SCP domain	Function unknown
**76117**	-146	-140	6	Defense-related protein containing SCP domain	Function unknown
**57609**	-1466	-1450	16	Heterochromatin-associated protein HP1 and related CHROMO domain proteins	Chromatin structure and dynamics
**105191**	-1220	-1190	30	RNA polymerase I and III, subunit RPA40/RPC40	Transcription
**57335**	-195	-172	23	Uncharacterized conserved protein	Function unknown
**52847**	-604	-589	15	NADH:ubiquinone oxidoreductase, NDUFA5/B13 subunit	Energy production and conversion
**124285**	-674	-665	9	Small nuclear ribonucleoprotein SMD1 and related snRNPs	RNA processing and modification
**105342**	-766	-749	17	von Willebrand factor and related coagulation proteins	Defense mechanisms / Extracellular structures
**76336**	-1434	-1428	6	Mitofusin 1 GTPase, involved in mitochondrila biogenesis	Posttranslational modification, protein turnover, chaperones
**105432**	-947	-938	9	FOG: Immunoglobin and related proteins	Inorganic ion transport and metabolism / General function prediction only
**59014**	-1217	-1211	6	Pleiotropic drug resistance proteins (PDR1-15), ABC superfamily	Secondary metabolites biosynthesis, transport and catabolism
**59014**	-1211	-1199	12	Pleiotropic drug resistance proteins (PDR1-15), ABC superfamily	Secondary metabolites biosynthesis, transport and catabolism
**59014**	-1199	-1193	6	Pleiotropic drug resistance proteins (PDR1-15), ABC superfamily	Secondary metabolites biosynthesis, transport and catabolism

**59014**	-1193	-1187	6	Pleiotropic drug resistance proteins (PDR1-15), ABC superfamily	Secondary metabolites biosynthesis, transport and catabolism
**58539**	-659	-653	6	Mitochondrial ribosome small subunit component, mediator of apoptosis DAP3	Translation, ribosomal structure and biogenesis
**121158**	-1320	-1297	23	Serine/threonine protein kinase	Signal transduction mechanisms
**105931**	-761	-755	6	Beta-N-acetylhexosaminidase	Carbohydrate transport and metabolism
**105931**	-755	-738	17	Beta-N-acetylhexosaminidase	Carbohydrate transport and metabolism
**105931**	-738	-721	17	Beta-N-acetylhexosaminidase	Carbohydrate transport and metabolism
**105931**	-721	-704	17	Beta-N-acetylhexosaminidase	Carbohydrate transport and metabolism
**105931**	-704	-680	24	Beta-N-acetylhexosaminidase	Carbohydrate transport and metabolism
**106051**	-819	-813	6	Transcription factor TCF20	Transcription
**106067**	-1137	-1130	7	Mitochondrial chaperonin	Posttranslational modification, protein turnover, chaperones
**47069**	-177	-165	12	3'-5' exonuclease	Replication, recombination and repair
**60346**	-85	-74	11	Para-aminobenzoate (PABA) synthase ABZ1	Translation, ribosomal structure and biogenesis
**60008**	-1339	-1318	21	Phosphatidylethanolamine binding protein	General function prediction only
**60873**	-84	-55	29	ATP-dependent DNA ligase I	Replication, recombination and repair
**61159**	-49	-39	10	Subunit of cis-Golgi transport vesicle tethering complex - Sec34p	Intracellular trafficking, secretion, and vesicular transport
**107187**	-1093	-1077	16	Reductases with broad range of substrate specificities	General function prediction only
**3889**	-1330	-1324	6	von Willebrand factor and related coagulation proteins	Defense mechanisms / Extracellular structures
**3889**	-1324	-1306	18	von Willebrand factor and related coagulation proteins	Defense mechanisms / Extracellular structures
**3889**	-1306	-1300	6	von Willebrand factor and related coagulation proteins	Defense mechanisms / Extracellular structures
**3889**	-1300	-1282	18	von Willebrand factor and related coagulation proteins	Defense mechanisms / Extracellular structures
**121777**	-1222	-1216	6	Halotolerance protein HAL3 (contains flavoprotein domain)	Cell cycle control, cell division, chromosome partitioning / Inorganic ion transport and metabolism
**107323**	-265	-251	14	Predicted 3-ketosphinganine reductase	Secondary metabolites biosynthesis, transport and catabolism
**121820**	-279	-270	9	Methionine synthase II (cobalamin-independent)	Amino acid transport and metabolism
**77991**	-578	-561	17	RNA polymerase II transcription initiation/nucleotide excision repair factor TFIIH, subunit SSL1	Transcription / Replication, recombination and repair
**78072**	-617	-596	21	Amidases	Translation, ribosomal structure and biogenesis
**121915**	-894	-884	10	Microtubule-binding protein (translationally controlled tumor protein)	Cell cycle control, cell division, chromosome partitioning / Cytoskeleton

**54991**	-893	-884	9	Hydroxysteroid 17-beta dehydrogenase 11	Secondary metabolites biosynthesis, transport and catabolism
**53437**	-305	-278	27	Putative RNA polymerase II regulator	Transcription
**44041**	-403	-394	9	NADH-ubiquinone oxidoreductase, NDUFS1/75 kDa subunit	Energy production and conversion
**102947**	-165	-135	30	Speckle-type POZ protein SPOP and related proteins with TRAF, MATH and BTB/POZ domains	Cell cycle control, cell division, chromosome partitioning / General function prediction only
**21324**	-1145	-1118	27	Protein phosphatase 2A regulatory subunit A and related proteins	Signal transduction mechanisms
**54144**	-187	-178	9	Ferric reductase, NADH/NADPH oxidase and related proteins	Inorganic ion transport and metabolism / Secondary metabolites biosynthesis, transport and catabolism
**53685**	-1412	-1405	7	Type I phosphodiesterase/nucleotide pyrophosphatase	General function prediction only
**33273**	-1174	-1165	9	Mitochondrial/chloroplast ribosomal protein L17	Translation, ribosomal structure and biogenesis
**103350**	-417	-396	21	Mitochondrial ribosomal protein S28	Translation, ribosomal structure and biogenesis
**4064**	-1321	-1311	10	Threonine/serine dehydratases	Amino acid transport and metabolism
**34197**	-1201	-1195	6	Stress-induced protein UVI31+	Signal transduction mechanisms
**62706**	-1095	-1089	6	Protein phosphatase 1, regulatory subunit, and related proteins	Signal transduction mechanisms
**62314**	-1329	-1323	6	Transcription initiation factor TFIID, subunit BDF1 and related bromodomain proteins	Transcription
**122124**	-899	-879	20	AAA+-type ATPase	Posttranslational modification, protein turnover, chaperones
**78582**	-723	-708	15	Protoheme ferro-lyase (ferrochelatase)	Coenzyme transport and metabolism
**4213**	-22	-11	11	Ribonuclease, T2 family	RNA processing and modification
**63206**	-1089	-1074	15	Putative zinc transporter	Inorganic ion transport and metabolism
**122240**	-337	-328	9	Carnitine O-acyltransferase CPT2/YAT1	Lipid transport and metabolism
**36727**	-511	-505	6	SWI-SNF chromatin-remodeling complex protein	Chromatin structure and dynamics
**78909**	-1333	-1324	9	NDR and related serine/threonine kinases	General function prediction only
**4475**	-743	-716	27	C-3 sterol dehydrogenase/3-beta-hydroxysteroid dehydrogenase and related dehydrogenases	Amino acid transport and metabolism / Lipid transport and metabolism
**63882**	-622	-613	9	Enoyl-CoA hydratase	Lipid transport and metabolism
**79244**	-1310	-1303	7	Serine/threonine protein kinase	Signal transduction mechanisms
**108783**	-1188	-1169	19	Flavin-containing monooxygenase	Secondary metabolites biosynthesis, transport and catabolism
**79324**	-107	-89	18	Flavonol reductase/cinnamoyl-CoA reductase	Defense mechanisms
**79329**	-306	-276	30	Permease of the major facilitator superfamily	General function prediction only

**79345**	-1160	-1151	9	Peroxisomal membrane protein MPV17 and related proteins	General function prediction only
**64545**	-391	-385	6	Monocarboxylate transporter	Carbohydrate transport and metabolism
**109007**	-953	-947	6	FOG: Low-complexity	Function unknown
**22785**	-604	-581	23	FOG: Zn-finger	General function prediction only
**22785**	-296	-290	6	FOG: Zn-finger	General function prediction only
**65142**	-84	-78	6	Aldehyde dehydrogenase	Energy production and conversion
**109397**	-1191	-1178	13	Uncharacterized conserved protein	Function unknown
**122886**	-1332	-1305	27	Beta tubulin	Cytoskeleton
**50335**	-1344	-1335	9	Ras-related small GTPase, Rho type	General function prediction only
**122988**	-488	-463	25	Multidrug resistance-associated protein/mitoxantrone resistance protein, ABC superfamily	Secondary metabolites biosynthesis, transport and catabolism
**65925**	-937	-914	23	Cytosine deaminase FCY1 and related enzymes	Nucleotide transport and metabolism
**123015**	-1243	-1213	30	GTPase Rab5/YPT51 and related small G protein superfamily GTPases	Intracellular trafficking, secretion, and vesicular transport
**123029**	-1445	-1437	8	Cu2+/Zn2+ superoxide dismutase SOD1	Inorganic ion transport and metabolism
**65965**	-350	-326	24	SAM-dependent methyltransferases	Lipid transport and metabolism / General function prediction only
**65821**	-1415	-1397	18	Uncharacterized conserved protein, contains WD40 repeats	Function unknown
**22911**	-64	-39	25	Glucose-repressible alcohol dehydrogenase transcriptional effector CCR4 and related proteins	Transcription
**66484**	-1140	-1116	24	Guanine nucleotide exchange factor	Intracellular trafficking, secretion, and vesicular transport
**5041**	-1238	-1211	27	Transcription initiation factor IIE, alpha subunit	Transcription
**123185**	-487	-481	6	Myosin class V heavy chain	Cytoskeleton
**66844**	-625	-619	6	Glycosylphosphatidylinositol anchor synthesis protein	Signal transduction mechanisms
**110158**	-1136	-1130	6	Predicted small molecule transporter involved in cellular pH homeostasis (Batten disease protein in human)	General function prediction only
**55912**	-1113	-1088	25	Septin CDC10 and related P-loop GTPases	Cell cycle control, cell division, chromosome partitioning / Signal transduction mechanisms / Cytoskeleton
**103470**	-1286	-1276	10	RNA-directed RNA polymerase QDE-1 required for posttranscriptional gene silencing and RNA interference	RNA processing and modification
**44878**	-1369	-1351	18	Arp2/3 complex-interacting protein VIP1/Asp1, involved in regulation of actin cytoskeleton	Cytoskeleton

**2358**	-1085	-1055	30	Diadenosine polyphosphate hydrolase and related proteins of the histidine triad (HIT) family	Nucleotide transport and metabolism / General function prediction only
**2365**	-1183	-1175	8	Putative N2,N2-dimethylguanosine tRNA methyltransferase	RNA processing and modification
**21505**	-945	-924	21	G-protein alpha subunit (small G protein superfamily)	Cell cycle control, cell division, chromosome partitioning / Signal transduction mechanisms
**75069**	-806	-782	24	Predicted membrane protein, contains DoH and Cytochrome b-561/ferric reductase transmembrane domains	Signal transduction mechanisms
**110636**	-1088	-1082	6	RRM motif-containing protein	RNA processing and modification
**80961**	-1208	-1197	11	Transcription factor TCF20	Transcription
**51212**	-808	-802	6	60S ribosomal protein L5	Translation, ribosomal structure and biogenesis
**67732**	-1034	-1017	17	Multidrug resistance-associated protein/mitoxantrone resistance protein, ABC superfamily	Secondary metabolites biosynthesis, transport and catabolism
**51455**	-113	-107	6	ATP-dependent RNA helicase	RNA processing and modification
**110949**	-1127	-1115	12	C-type lectin	Defense mechanisms / Signal transduction mechanisms
**68122**	-772	-755	17	Permease of the major facilitator superfamily	Carbohydrate transport and metabolism
**111192**	-1420	-1411	9	Nucleolar GTPase/ATPase p130	Nuclear structure
**111447**	-1281	-1270	11	Sulfatase	General function prediction only
**23276**	-978	-969	9	VAMP-associated protein involved in inositol metabolism	Intracellular trafficking, secretion, and vesicular transport
**81822**	-491	-461	30	Leucyl-tRNA synthetase	Translation, ribosomal structure and biogenesis
**69651**	-454	-443	11	Predicted transporter (major facilitator superfamily)	General function prediction only
**75195**	-438	-432	6	NADPH oxidase	Energy production and conversion
**75195**	-432	-420	12	NADPH oxidase	Energy production and conversion
**120384**	-1062	-1035	27	Notchless-like WD40 repeat-containing protein	Function unknown
**75383**	-1447	-1441	6	Reductases with broad range of substrate specificities	General function prediction only
**75403**	-642	-620	22	Cysteine desulfurase NFS1	Amino acid transport and metabolism
**104418**	-84	-78	6	Kynurenine 3-monooxygenase and related flavoprotein monooxygenases	Energy production and conversion / General function prediction only
**75551**	-1201	-1174	27	Predicted sugar kinase	Carbohydrate transport and metabolism
**104593**	-166	-157	9	FOG: WD40 repeat	General function prediction only
**52521**	-1252	-1246	6	Uncharacterized conserved protein	Function unknown

**6011**	-538	-514	24	Phosphatidic acid-preferring phospholipase A1, contains DDHD domain	Intracellular trafficking, secretion, and vesicular transport / Lipid transport and metabolism
**57870**	-690	-684	6	Histones H3 and H4	Chromatin structure and dynamics
**104785**	-1099	-1085	14	Phosphoinositide phosphatase SAC1	Lipid transport and metabolism
**75845**	-1442	-1431	11	Cysteine desulfurase NFS1	Amino acid transport and metabolism
**45980**	-1431	-1424	7	Cell cycle control protein	Transcription / Cell cycle control, cell division, chromosome partitioning / Signal transduction mechanisms
**57472**	-201	-187	14	FOG: RRM domain	General function prediction only
**76057**	-761	-753	8	Vacuolar H+-ATPase V1 sector, subunit B	Energy production and conversion
**58011**	-620	-611	9	FOG: Zn-finger	General function prediction only
**57286**	-1396	-1373	23	Histone H3 (Lys4) methyltransferase complex, subunit CPS25/DPY-30	Transcription
**82651**	-1170	-1161	9	Cullins	Cell cycle control, cell division, chromosome partitioning
**46463**	-968	-962	6	Serine palmitoyltransferase	Posttranslational modification, protein turnover, chaperones
**46613**	-161	-155	6	Predicted membrane protein	Function unknown
**76700**	-108	-81	27	FOG: Zn-finger	General function prediction only / Transcription
**105968**	-1071	-1044	27	Iron/ascorbate family oxidoreductases	Secondary metabolites biosynthesis, transport and catabolism / General function prediction only
**76905**	-1230	-1203	27	Flavin-containing monooxygenase	Secondary metabolites biosynthesis, transport and catabolism
**121298**	-1175	-1145	30	Glyoxylate/hydroxypyruvate reductase (D-isomer-specific 2-hydroxy acid dehydrogenase superfamily)	Energy production and conversion
**121441**	-702	-672	30	Synaptic vesicle transporter SVOP and related transporters (major facilitator superfamily)	General function prediction only
**121529**	-1252	-1227	25	Mitochondrial carrier protein MRS3/4	Energy production and conversion
**77495**	-1064	-1035	29	Uncharacterized conserved protein	Function unknown
**107014**	-248	-239	9	Nucleolar GTPase/ATPase p130	Nuclear structure
**121786**	-1182	-1168	14	Uncharacterized conserved protein	Function unknown
**62493**	-384	-372	12	Chromatin remodeling complex RSC, subunit RSC1/Polybromo and related proteins	Transcription / Chromatin structure and dynamics
**27939**	-964	-953	11	Predicted transporter (major facilitator superfamily)	General function prediction only
**70750**	-1410	-1395	15	Vacuolar H+-ATPase V0 sector, subunit M9.7 (M9.2)	Energy production and conversion
**29619**	-113	-83	30	Annexin	Intracellular trafficking, secretion, and vesicular transport

**34112**	-1179	-1166	13	60S ribosomal protein L22	Translation, ribosomal structure and biogenesis
**69217**	-1144	-1121	23	Cdk activating kinase (CAK)/RNA polymerase II transcription initiation/nucleotide excision repair factor TFIIH/TFIIK, kinase subunit CDK7	Transcription / Replication, recombination and repair / Cell cycle control, cell division, chromosome partitioning
**30465**	-158	-152	6	U4/U6 small nuclear ribonucleoprotein Prp4 (contains WD40 repeats)	RNA processing and modification
**56872**	-273	-264	9	Serine/threonine specific protein phosphatase involved in glycogen accumulation, PP2A-related	Signal transduction mechanisms / Carbohydrate transport and metabolism
**55969**	-1040	-1030	10	Translation initiation factor 1A (eIF-1A)	Translation, ribosomal structure and biogenesis
**54471**	-320	-295	25	Serine/threonine protein kinase	Signal transduction mechanisms
**59546**	-1191	-1184	7	Transcription coactivator	Transcription
**41035**	-1398	-1389	9	Calcium-responsive transcription coactivator	Transcription
**69013**	-1375	-1369	6	Uncharacterized conserved protein	Function unknown
**69013**	-1369	-1363	6	Uncharacterized conserved protein	Function unknown
**33704**	-835	-822	13	Transcription initiation factor TFIID, subunit TAF9 (also component of histone acetyltransferase SAGA)	Transcription
**27649**	-767	-742	25	FOG: Zn-finger	General function prediction only
**63092**	-1377	-1360	17	Ribulose kinase and related carbohydrate kinases	Carbohydrate transport and metabolism
**25244**	-1165	-1136	29	FOG: Zn-finger	General function prediction only
**61032**	-364	-354	10	FOG: RCC1 domain	Function unknown
**39285**	-405	-399	6	Predicted DNA methylase	Function unknown
**55114**	-1056	-1029	27	Nucleoside phosphatase	Nucleotide transport and metabolism
**54736**	-776	-770	6	ATP-dependent RNA helicase	RNA processing and modification
**103131**	-597	-591	6	unknown protein	-
**53596**	-505	-489	16	unknown protein	-
**78189**	-218	-212	6	unknown protein	-
**62386**	-257	-246	11	unknown protein	-
**62537**	-1270	-1258	12	unknown protein	-
**62333**	-1237	-1211	26	unknown protein	-
**71532**	-715	-686	29	unknown protein	-

**108025**	-1294	-1270	24	unknown protein	-
**4221**	-983	-968	15	unknown protein	-
**108202**	-245	-236	9	unknown protein	-
**108202**	-236	-227	9	unknown protein	-
**108573**	-574	-565	9	unknown protein	-
**108683**	-1011	-981	30	unknown protein	-
**79222**	-225	-198	27	unknown protein	-
**79222**	-198	-171	27	unknown protein	-
**79222**	-121	-107	14	unknown protein	-
**122576**	-1287	-1281	6	unknown protein	-
**79921**	-755	-745	10	unknown protein	-
**65572**	-201	-182	19	unknown protein	-
**80252**	-1433	-1409	24	unknown protein	-
**5011**	-622	-616	6	unknown protein	-
**123186**	-324	-318	6	unknown protein	-
**110159**	-978	-972	6	unknown protein	-
**110825**	-683	-674	9	unknown protein	-
**68026**	-841	-835	6	unknown protein	-
**81586**	-424	-411	13	unknown protein	-
**81586**	-411	-405	6	unknown protein	-
**81586**	-405	-399	6	unknown protein	-
**81586**	-399	-393	6	unknown protein	-
**81586**	-393	-387	6	unknown protein	-
**68876**	-1402	-1389	13	unknown protein	-
**68876**	-1389	-1383	6	unknown protein	-
**68876**	-1383	-1377	6	unknown protein	-
**68876**	-1377	-1371	6	unknown protein	-
**68876**	-1371	-1365	6	unknown protein	-

**111439**	-1420	-1402	18	unknown protein	-
**68948**	-1395	-1389	6	unknown protein	-
**68948**	-1389	-1383	6	unknown protein	-
**123955**	-59	-50	9	unknown protein	-
**81979**	-1125	-1116	9	unknown protein	-
**104071**	-1404	-1398	6	unknown protein	-
**104419**	-17	-11	6	unknown protein	-
**56218**	-428	-400	28	unknown protein	-
**41699**	-920	-896	24	unknown protein	-
**57534**	-660	-653	7	unknown protein	-
**75935**	-879	-869	10	unknown protein	-
**105176**	-68	-62	6	unknown protein	-
**59073**	-774	-767	7	unknown protein	-
**58282**	-1373	-1364	9	unknown protein	-
**121091**	-1179	-1154	25	unknown protein	-
**106154**	-323	-296	27	unknown protein	-
**121337**	-741	-735	6	unknown protein	-
**106452**	-923	-902	21	unknown protein	-
**106452**	-726	-720	6	unknown protein	-
**60578**	-1093	-1087	6	unknown protein	-
**60422**	-21	-12	9	unknown protein	-
**107111**	-38	-19	19	unknown protein	-
**48178**	-71	-41	30	unknown protein	-
**54352**	-1375	-1369	6	unknown protein	-
**54426**	-343	-334	9	unknown protein	-
**103130**	-965	-959	6	unknown protein	-
**62198**	-1023	-994	29	unknown protein	-
**78706**	-1025	-1016	9	unknown protein	-

**78706**	-1016	-1007	9	unknown protein	-
**78706**	-197	-176	21	unknown protein	-
**71676**	-231	-222	9	unknown protein	-
**63648**	-453	-444	9	unknown protein	-
**43418**	-1062	-1043	19	unknown protein	-
**65808**	-1246	-1240	6	unknown protein	-
**123256**	-1330	-1321	9	unknown protein	-
**103563**	-1396	-1390	6	unknown protein	-
**80685**	-1479	-1472	7	unknown protein	-
**80685**	-1472	-1465	7	unknown protein	-
**80685**	-1465	-1458	7	unknown protein	-
**67035**	-958	-950	8	unknown protein	-
**81149**	-1475	-1461	14	unknown protein	-
**51907**	-1468	-1439	29	unknown protein	-
**72488**	-369	-356	13	unknown protein	-
**56531**	-703	-691	12	unknown protein	-
**120407**	-1136	-1127	9	unknown protein	-
**75394**	-282	-259	23	unknown protein	-
**56259**	-530	-508	22	unknown protein	-
**111991**	-603	-594	9	unknown protein	-
**111991**	-594	-588	6	unknown protein	-
**111991**	-588	-570	18	unknown protein	-
**112212**	-223	-196	27	unknown protein	-
**105389**	-403	-383	20	unknown protein	-
**105520**	-761	-755	6	unknown protein	-
**105520**	-755	-743	12	unknown protein	-
**105520**	-743	-737	6	unknown protein	-
**105520**	-737	-731	6	unknown protein	-

**105524**	-995	-986	9	unknown protein	-
**58634**	-744	-738	6	unknown protein	-
**121355**	-1442	-1414	28	unknown protein	-
**106444**	-1374	-1358	16	unknown protein	-
**60004**	-1409	-1388	21	unknown protein	-
**60004**	-1212	-1206	6	unknown protein	-
**60897**	-234	-220	14	unknown protein	-
**60665**	-1234	-1224	10	unknown protein	-
**3739**	-1305	-1288	17	unknown protein	-
**121638**	-177	-171	6	unknown protein	-
**32204**	-29	-14	15	unknown protein	-
**33058**	-769	-757	12	unknown protein	-
**59703**	-1203	-1178	25	unknown protein	-
**66865**	-83	-65	18	unknown protein	-
**55698**	-960	-950	10	unknown protein	-
**34353**	-1035	-1029	6	unknown protein	-
**65817**	-1389	-1383	6	unknown protein	-
**32293**	-815	-792	23	unknown protein	-
**32293**	-93	-69	24	unknown protein	-
**42953**	-794	-776	18	unknown protein	-
**26163**	-291	-270	21	unknown protein	-

*Positions of Sites 1 and 2 are relative to the gene start codon.
