# Supplementary material for: Deciphering the Cis-Regulatory Elements for XYR1 and CRE1 Regulators in Trichoderma reesei
Source: PLoS One. 2014 Jun 18;9(6):e99366. doi: 10.1371/journal.pone.0099366 (PMC4062390; doi:10.1371/journal.pone.0099366)
Supplement: Tables S6 — Prediction of XYR1 binding sites on genes down regulated in a Δxyr1 mutant induced with cellulose. (PDF) [file pone.0099366.s006.pdf]

Tables S6. Prediction of XYR1 binding sites on genes down regulated in a *Δxyr1* mutant induced with cellulose.

Protein ID	Site 1*	Site 2	Distance	Description	log2 FC (Δxyr1/wt)	p-val
**76210**	-614	-590	24	GH62 α-L-arabinofuranosidase ABF2	-8.404019511	8.93E-100
**76682**	-1180	-1170	10	PDR-type ABC transporters	-5.425185968	0.004432593
**112031**	-272	-246	26	unique protein, secreted	-4.786445676	0.000000157
**103012**	-1366	-1338	28	taurine catabolism dioxygenase, alpha-ketoglutarate dependent	-4.392656507	4.1E-82
**103012**	-1338	-1322	16	taurine catabolism dioxygenase, alpha-ketoglutarate dependent	-4.392656507	4.1E-82
**68230**	-390	-376	14	N-methylhydantoinase A/acetone carboxylase HyuA, beta subunit	-4.287190072	0.00000867
**110173**	-426	-409	17	unknown protein	-3.825652012	3.29E-20
**68831**	-640	-612	28	GT α-1.2-mannosyltransferase	-3.772809949	3.01E-26
**82026**	-1077	-1049	28	trans-aconitate methyltransferase	-3.502482697	3.69E-10
**123251**	-995	-980	15	flavohemoglobin	-3.084271181	0.00000221
**109234**	-1325	-1295	30	D-aminopeptidase	-3.029118614	1.84E-45
**122778**	-1050	-1039	11	short chain dehydrogenase/reductase	-2.972230198	0.00000107
**64959**	-1145	-1134	11	phosphatidyl synthase	-2.86576707	0.000283823
**121664**	-641	-624	17	Glutamate decarboxylase and related proteins	-2.660868678	8.84E-27
**121664**	-203	-182	21	Glutamate decarboxylase and related proteins	-2.660868678	8.84E-27
**77299**	-1013	-998	15	GH2 Exo-β-D-glucosaminidase GLS93	-2.653128246	5.94E-32
**70803**	-745	-734	11	bifunctional catalase/peroxidase	-2.565881792	1.43E-41
**82235**	-240	-228	12	GH31 α-glucosidase	-2.382019398	7.48E-36
**80019**	-1278	-1248	30	short-chain dehydrogenase/reductase	-2.346522643	8.22E-36
**122416**	-1100	-1079	21	NADH:flavin oxidoreductase/NADH oxidase	-2.191215477	0.021716439
**44362**	-637	-613	24	unknown protein	-2.188442554	1.32E-11
**54768**	-1135	-1124	11	unknown protein	-2.023759917	2.82E-09
**67035**	-1260	-1231	29	unknown protein	-1.895382889	0.009207258
**51499**	-647	-618	29	ketol-acid reductoisomerase. mitochondrial precursor	-1.754031926	2.86E-20

**121605**	-459	-431	28	unknown protein	-1.692958087	0.0000635
**121082**	-825	-804	21	unique protein	-1.681533485	2.57E-10
**121894**	-243	-232	11	Zn2Cys6 transcriptional regulator	-1.681050265	8.37E-17
**39637**	-880	-863	17	C4-dicarboxylate transporter/malic acid transport protein	-1.646295211	0.000000231
**82667**	-749	-720	29	ThrB Homoserine kinase	-1.641776897	1.19E-12
**73924**	-346	-332	14	MDR-type ABC transporters	-1.616223561	4.9E-17
**45445**	-271	-256	15	tyrosinase	-1.597808419	1.94E-17
**45445**	-256	-235	21	tyrosinase	-1.597808419	1.94E-17
**123614**	-370	-347	23	unknown protein	-1.594060959	8.07E-18
**108459**	-922	-906	16	carbamoyl-phosphate synthase (glutamine-hydrolyzing)arginine-specific large chain	-1.543820058	2E-16
**68254**	-618	-604	14	Zn2Cys6 transcriptional regulator	-1.531287544	0.002243848
**4213**	-587	-565	22	ribonuclease T2	-1.49489293	1.63E-10
**108586**	-1227	-1217	10	unknown protein	-1.48693498	0.0000444
**65711**	-1393	-1371	22	SAM-dependent methyltransferase	-1.483172411	2.08E-12
**70025**	-681	-658	23	alcohol dehydrogenase. zinc-containing. putative	-1.463905484	1.64E-08
**123473**	-725	-697	28	MFS permease	-1.45458798	1.14E-10
**121826**	-454	-434	20	ATP citrate lyase. beta subunit	-1.403597391	2.05E-13
**119902**	-1065	-1040	25	unique protein	-1.388942427	0.000574214
**124282**	-1339	-1324	15	unknown protein. SET and MYND domain	-1.38125509	0.000022
**61750**	-1070	-1056	14	Sur2 sphinganine C4-hydroxylase of Saccharomyces cerevisiae	-1.319795	0.001902816
**61750**	-105	-76	29	Sur2 sphinganine C4-hydroxylase of Saccharomyces cerevisiae	-1.319795	0.001902816
**122920**	-1098	-1083	15	Molecular chaperone Bip	-1.315108954	3.07E-11
**122920**	-1083	-1062	21	Molecular chaperone Bip	-1.315108954	3.07E-11
**78496**	-1355	-1333	22	Flavin-containing monooxygenase Fmo1 like	-1.29700035	4.25E-11
**78496**	-1333	-1323	10	Flavin-containing monooxygenase Fmo1 like	-1.29700035	4.25E-11
**49753**	-800	-790	10	L-arabinitol 4-dehydrogenase	-1.291016577	7.14E-09
**72339**	-383	-370	13	GH18. chitinase CHI18-9	-1.277106166	9.42E-09
**122579**	-499	-481	18	unknown protein. 4TM	-1.272554104	3.68E-09

**122579**	-481	-468	13	unknown protein. 4TM	-1.272554104	3.68E-09
**72643**	-1338	-1324	14	arginosuccinate synthetase	-1.271628185	7.45E-11
**77481**	-1179	-1168	11	D-xylulose 5-phosphate/D-fructose 6-phosphate phosphoketolase	-1.266046924	0.034863148
**77481**	-1168	-1152	16	D-xylulose 5-phosphate/D-fructose 6-phosphate phosphoketolase	-1.266046924	0.034863148
**123131**	-167	-150	17	endonuclease/exonuclease/phosphatase. putative	-1.239646639	0.00000104
**120008**	-1332	-1317	15	GMC oxidoreductase	-1.183531098	0.000000002
**49649**	-1334	-1324	10	unknown protein	-1.154376595	1.43E-08
**107670**	-1108	-1088	20	unknown protein	-1.137040128	0.027299852
**119609**	-1103	-1093	10	Phospho-2-dehydro-3-deoxyheptonate aldolase	-1.134155176	5.67E-08
**120837**	-836	-817	19	unknown protein. 1 TM domain	-1.132484858	5.68E-10
**53246**	-438	-423	15	unknown protein	-1.110617242	3.86E-09
**111994**	-496	-482	14	unknown protein	-1.087373342	0.000000436
**80058**	-21	-9	12	MFS permease	-1.027646883	0.0000405
**69171**	-258	-247	11	unknown protein	-1.012296116	0.036441159
**75589**	-376	-360	16	hydroxymethylglutaryl CoA synthase	-1.006759852	0.00000252
**124113**	-677	-664	13	PTH11 GPCR	-1.004694118	0.000000122

*Positions of Sites 1 and 2 are relative to the gene start codon.
