# Supplementary material for: Deciphering the Cis-Regulatory Elements for XYR1 and CRE1 Regulators in Trichoderma reesei
Source: PLoS One. 2014 Jun 18;9(6):e99366. doi: 10.1371/journal.pone.0099366 (PMC4062390; doi:10.1371/journal.pone.0099366)
Supplement: Tables S7 — Prediction of XYR1 binding sites on genes up regulated in a Δxyr1 mutant induced with cellulose. (PDF) [file pone.0099366.s007.pdf]

Tables S7. Prediction of XYR1 binding sites on genes up regulated in a *Δxyr1* mutant induced with cellulose.

Protein ID	Site 1*	Site 2	Distance	Description	log2 FC (Δxyr1/wt)	p-val
**124175**	-1324	-1299	25	GH64 endo-1,3-β-glucanase	3.536934872	0.001695692
**61142**	-302	-280	22	unknown protein	2.942067817	1.72E-41
**57558**	-727	-703	24	unknown protein	2.93145646	1.33E-33
**109278**	-380	-358	22	GH24 lysozyme	2.691119136	1.21E-10
**123865**	-1491	-1479	12	Peptidase S8 and S53, subtilisin, kexin, sedolisin	2.673350404	3.64E-09
**29716**	-832	-804	28	thiamine-repressible mitochondrial transport protein THI74, putative	2.44392262	5.87E-39
**104180**	-1410	-1384	26	Ankyrin	2.418346823	2.28E-09
**123911**	-609	-583	26	unknown protein	2.32803859	1.11E-22
**40918**	-1150	-1132	18	unknown protein	2.064471614	1.6E-26
**106457**	-830	-811	19	unknown protein	2.033383557	2.2E-26
**70452**	-1411	-1393	18	flavin containing amine oxidoreductase	2.028150258	4.97E-15
**70452**	-1393	-1373	20	flavin containing amine oxidoreductase	2.028150258	4.97E-15
**67484**	-74	-58	16	AMP deaminase	1.94091069	2.59E-18
**5007**	-1080	-1051	29	unknown protein	1.940907236	3.12E-08
**62244**	-1385	-1369	16	Zn2Cys6 transcriptional regulator	1.933640298	2.12E-18
**64018**	-1010	-993	17	GPCR, mating type pheromone G-protein coupled receptor	1.898127037	8.32E-19
**120224**	-142	-125	17	C2H2 transcriptional regulator	1.849624272	2.57E-23
**123658**	-343	-331	12	unique protein	1.796126706	0.00000175
**43129**	-492	-465	27	unknown protein	1.787519363	0.000576368
**123241**	-772	-755	17	MFS permease	1.763532244	7.74E-09
**65921**	-226	-197	29	Acetyl/propionyl-CoA carboxylase alpha subunit	1.62109277	8.08E-11
**65921**	-197	-177	20	Acetyl/propionyl-CoA carboxylase alpha subunit	1.62109277	8.08E-11
**47603**	-972	-960	12	succinyl-CoA:3-ketoacid-coenzyme A transferase subunit A	1.597300255	0.000000717
**121839**	-1277	-1261	16	HSP70	1.471560323	3.36E-09

**65615**	-696	-679	17	RibosomalRNA methyltransferaseRrmJ/FtsJ domain.	1.470196019	5.02E-12
**53067**	-1270	-1260	10	Zn2Cys6 transcriptional regulator	1.46394108	1.17E-12
**104469**	-1193	-1177	16	unknown protein	1.460363146	3.8E-16
**65821**	-450	-437	13	vacuolar targeting protein Atg18	1.38188595	8.63E-13
**81756**	-309	-281	28	unknown protein	1.313582505	0.0000576
**61819**	-743	-717	26	unknown protein	1.306390838	4.39E-08
**111253**	-1093	-1069	24	unknown protein	1.306340446	2.41E-08
**111253**	-621	-611	10	unknown protein	1.306340446	2.41E-08
**23455**	-534	-519	15	unknown protein	1.276813859	6.15E-11
**55679**	-85	-69	16	unknown protein	1.272451846	1.01E-10
**123508**	-38	-27	11	unknown protein	1.242351576	3.87E-09
**69611**	-1217	-1188	29	MFS permease	1.233214194	3.67E-08
**69611**	-246	-231	15	MFS permease	1.233214194	3.67E-08
**76151**	-517	-493	24	GT31 ß-glycosyltransferase	1.225525667	6.35E-10
**111446**	-1316	-1295	21	Zn2Cys6 transcriptional regulator	1.189224202	0.00057895
**111216**	-1352	-1332	20	histone H3 methyltransferase	1.175584913	1.81E-08
**67579**	-894	-868	26	phospholipase A2	1.158968279	0.000000196
**107715**	-1090	-1060	30	SSCRP	1.148333876	0.002529392
**55005**	-768	-756	12	GT22 ALG9 mannosyltransferase	1.122654079	4.04E-08
**23190**	-128	-107	21	mRNA cleavage factor complex II protein Clp1, putative	1.101035304	1.67E-08
**121065**	-848	-835	13	unknown protein	1.092209544	0.000000106
**49873**	-942	-916	26	transcriptional regulator, unknown	1.071764481	1.63E-08
**104333**	-665	-653	12	unknown protein	1.063570219	0.000000459
**74731**	-468	-438	30	beta-adaptin, apl1, large subunit of the adaptor protein complex (AP-2) of clathrin-coated vesicles	1.051308628	2.31E-08
**59778**	-273	-256	17	GPCR, related to A nidulans GprC	1.042224749	0.0000014
**57513**	-89	-65	24	MAPKK, mitogen activated protein kinase kinase Ssk2p	1.018806038	0.000000282

*Positions of Sites 1 and 2 are relative to the gene start codon.
