# Supplementary material for: Deciphering the Cis-Regulatory Elements for XYR1 and CRE1 Regulators in Trichoderma reesei
Source: PLoS One. 2014 Jun 18;9(6):e99366. doi: 10.1371/journal.pone.0099366 (PMC4062390; doi:10.1371/journal.pone.0099366)
Supplement: Tables S8 — Prediction of XYR1 binding sites on genes down regulated in a Δxyr1 mutant induced with sophorose. (PDF) [file pone.0099366.s008.pdf]

Tables S8. Prediction of XYR1 binding sites on genes down regulated in a *Δxyr1* mutant induced with sophorose.

Protein ID	Site 1*	Site 2	Distance	Description	log2 FC (Δxyr1/wt)	p-val
**123261**	-939	-920	19	unknown protein	-2.99670971	1.28E-33
**123261**	-742	-732	10	unknown protein	-2.99670971	1.28E-33
**123261**	-681	-663	18	unknown protein	-2.99670971	1.28E-33
**44278**	-860	-847	13	Rab geranyl transferase escort protein	-2.120576253	3.95E-17
**123084**	-68	-43	25	chloroperoxidase	-1.85817748	0.003089378
**119860**	-1391	-1379	12	unknown protein	-1.797021174	7.57E-13
**120943**	-296	-270	26	NAD-dependent glutamate dehydrogenase	-1.788918503	1.01E-10
**121121**	-83	-56	27	Zn2Cys6 transcriptional regulator	-1.772560265	5.13E-11
**21960**	-1147	-1130	17	phospholipase C	-1.682812431	9.23E-10
**81756**	-309	-281	28	unknown protein	-1.59792776	6.06E-12
**109317**	-150	-126	24	unknown protein	-1.585583444	6.11E-10
**79960**	-907	-882	25	GH47 α-1,2-mannosidase	-1.442723348	1.2E-09
**56780**	-91	-72	19	DNA polymerase X family	-1.418313101	3.64E-08
**122043**	-224	-206	18	unknown protein	-1.379370847	4.21E-09
**58381**	-1364	-1337	27	unknown protein	-1.372650179	1.16E-08
**58381**	-1337	-1318	19	unknown protein	-1.372650179	1.16E-08
**122724**	-1106	-1078	28	unknown protein, only in neurospora and Chaetomium	-1.351846176	0.000000751
**123865**	-1491	-1479	12	Peptidase S8 and S53, subtilisin, kexin, sedolisin	-1.337624888	0.00000273
**122076**	-785	-773	12	aspartyl protease	-1.283009181	0.000000238
**122076**	-773	-746	27	aspartyl protease	-1.283009181	0.000000238
**103063**	-1332	-1312	20	unknown protein	-1.20931519	0.00000494
**75394**	-1321	-1310	11	unknown protein	-1.198644528	0.010961175
**121065**	-848	-835	13	unknown protein	-1.183685907	0.00000701
**121839**	-1277	-1261	16	HSP70	-1.156469003	0.0000016

**36391**	-89	-77	12	C2H2 transcriptional regulator	-1.127299804	0.00000192
**104219**	-455	-426	29	unknown protein	-1.118833451	0.022674252
**120224**	-142	-125	17	C2H2 transcriptional regulator	-1.116675465	0.0000031
**65921**	-226	-197	29	Acetyl/propionyl-CoA carboxylase alpha subunit	-1.088095997	0.0000587
**65921**	-197	-177	20	Acetyl/propionyl-CoA carboxylase alpha subunit	-1.088095997	0.0000587
**121107**	-1258	-1246	12	Zn2Cys6 transcriptional regulator	-1.085030781	0.00000534
**121107**	-94	-74	20	Zn2Cys6 transcriptional regulator	-1.085030781	0.00000534
**111103**	-223	-208	15	unknown protein	-1.079135101	0.00000389
**53596**	-338	-328	10	unknown protein	-1.078876955	0.0000233
**68291**	-1167	-1137	30	pyruvate formate lyase activating enzyme (radical SAM superfamily)	-1.067242861	0.000045
**47603**	-972	-960	12	succinyl-CoA:3-ketoacid-coenzyme A transferase subunit A	-1.055195306	0.0000152
**57322**	-165	-155	10	ARO Transcriptional regulators containing a DNA-binding HTH domain and an aminotransferase domain (MocR family)	-1.043339166	0.0000907

*Positions of Sites 1 and 2 are relative to the gene start codon.
