# Supplementary material for: Deciphering the Cis-Regulatory Elements for XYR1 and CRE1 Regulators in Trichoderma reesei
Source: PLoS One. 2014 Jun 18;9(6):e99366. doi: 10.1371/journal.pone.0099366 (PMC4062390; doi:10.1371/journal.pone.0099366)
Supplement: Tables S9 — Prediction of XYR1 binding sites on genes up regulated in a Δxyr1 mutant induced with sophorose. (PDF) [file pone.0099366.s009.pdf]

Tables S9. Prediction of XYR1 binding sites on genes up regulated in a *Δxyr1* mutant induced with sophorose.

Protein ID	Site 1*	Site 2	Distance	Description	log2 FC (Δxyr1/wt)	p-val
**52315**	-211	-201	10	copper transporter Ctr	9.504438019	2.73E-35
**111094**	-116	-93	23	CBM 13	6.543550884	2.49E-75
**82321**	-980	-956	24	Aquaporin	4.449578196	8.05E-29
**82321**	-878	-861	17	Aquaporin	4.449578196	8.05E-29
**82321**	-543	-526	17	Aquaporin	4.449578196	8.05E-29
**58717**	-431	-403	28	ß-lactamase class C	3.919694582	0.000000482
**123777**	-91	-73	18	unique protein	2.661861668	2.36E-10
**2316**	-560	-547	13	unknown protein with caleosin domain	2.28910924	0.000472964
**2316**	-547	-519	28	unknown protein with caleosin domain	2.28910924	0.000472964
**82327**	-161	-151	10	PDR-type ABC transporters	2.021735018	0.011071885
**106039**	-1022	-994	28	Ribosomal protein S28 based on homology to the corresponding protein of Neurospora crassa.	1.956909629	0.003884709
**106039**	-517	-489	28	Ribosomal protein S28 based on homology to the corresponding protein of Neurospora crassa.	1.956909629	0.003884709
**79238**	-1149	-1129	20	unknown protein	1.766548107	2.06E-10
**2451**	-1437	-1414	23	Secretion related small GTPase Rab6/Ypt6/Ryh1	1.73915014	1.04E-12
**78233**	-712	-684	28	40s ribosomal protein S21 based on homolgoy to the corresponding protein in Giberella zeae.	1.646507965	0.000419782
**34306**	-887	-857	30	mitochondrial cytochrome c oxidase assembly factor, putative	1.590507454	5.83E-08
**120983**	-939	-922	17	uracil phosphoribosyl transferase	1.563816786	0.0000831
**54768**	-1135	-1124	11	unknown protein	1.557910829	0.00419456
**105263**	-1264	-1254	10	Zn2Cys6 transcriptional regulator	1.528914054	2.13E-08
**68107**	-1497	-1475	22	Ribosomal protein S30 by homology with the corresponding protein of Ashbya gossypii.	1.513847592	0.001069802
**123176**	-754	-731	23	60S ribosomal protein L12 (L11 family).	1.439191543	0.010242256
**81930**	-166	-139	27	HAM1-like protein, probably related to DNA-repair	1.411901826	0.000000243
**120621**	-661	-649	12	ribosomal protein L37 of the large (60S) ribosomal subunit. Belongs to the same family as	1.404554321	2.43E-09

				yeast Rpl33Ap and has similarity to rat L35a.		
**105003**	-1390	-1365	25	unknown protein	1.40290366	3.52E-08
**123773**	-863	-851	12	Ubiquitin-conjugating enzyme	1.382287706	3.96E-09
**103147**	-1478	-1459	19	unknown protein	1.339385207	1.18E-08
**53947**	-1259	-1248	11	HFBs	1.33236182	6.5E-09
**119989**	-380	-354	26	HFB2	1.327482056	0.001596848
**121903**	-74	-53	21	iron-sulfur cluster assembly accessory protein, putative	1.316702511	0.000000156
**80206**	-705	-688	17	unknown protein	1.27696843	0.00000183
**21170**	-599	-582	17	Ribosomal protein S12	1.261659674	0.0000679
**71559**	-1435	-1415	20	Translation initiation factor eIF-5A gene based on homologyToThe corresponding genes in several filamentous fungi.	1.230204974	0.000000141
**71559**	-672	-645	27	Translation initiation factor eIF-5A gene based on homologyToThe corresponding genes in several filamentous fungi.	1.230204974	0.000000141
**103487**	-140	-123	17	glutathione S transferase	1.198183712	0.009117042
**79324**	-953	-937	16	nucleoside-diphosphate-sugar epimerase, putative (check!)	1.196939533	0.002519593
**40775**	-1386	-1362	24	RTA1 protein, 7 TNM, responds to xenobiotic stimulus	1.170915772	0.00000239
**74580**	-543	-514	29	unknown protein, 3 TM domains	1.153726051	0.000000704
**64874**	-892	-865	27	MFS toxin efflux pump	1.122511743	0.002675252
**49308**	-741	-711	30	unknown protein	1.106942966	0.001806411
**123202**	-44	-22	22	60s ribosomal protein rla2	1.100430395	0.035956414
**80709**	-672	-642	30	pre-mRNA-splicing factor CWC21, putative	1.09788588	0.000504897
**75447**	-1346	-1324	22	unknown protein	1.095846806	0.000012
**121003**	-1393	-1377	16	Ribosomal protein S18, S13 family	1.080354899	0.000103867
**49649**	-1334	-1324	10	unknown protein	1.080293983	0.006004053
**72643**	-1338	-1324	14	arginosuccinate synthetase	1.07930415	0.049072183
**74807**	-661	-633	28	GH76 GPI-anchored α-1,6-mannanase	1.067670775	0.00000811
**74807**	-601	-580	21	GH76 GPI-anchored α-1,6-mannanase	1.067670775	0.00000811
**120184**	-658	-646	12	v-SNARE Ykt6; trafficking to and within Golgi, endocytic trafficking to vacuole, vacuolar fusion	1.061753909	0.0000164
**60768**	-1129	-1112	17	unknown protein	1.061479399	0.0000555

**81541**	-1157	-1129	28	unknown protein	1.060246101	0.00000798
**121449**	-638	-627	11	IGPDImidazoleglycerol-phosphate dehydratase	1.057590989	0.0000797
**121620**	-252	-240	12	NMT1 thiamine biosynthesis protein	1.043177408	0.0000188
**80115**	-1402	-1372	30	unknown protein	1.034816624	0.027880534
**63813**	-319	-290	29	cytochrome b5, putative	1.032997124	0.00022569
**123659**	-859	-842	17	cell wall protein, istantly related to S. cerevisiae Pir3p.	1.023034243	0.001376401
**21396**	-83	-63	20	sugar isomerase	1.020934128	0.003012859

*Positions of Sites 1 and 2 are relative to the gene start codon.
