# Supplementary material for: Deciphering the Cis-Regulatory Elements for XYR1 and CRE1 Regulators in Trichoderma reesei
Source: PLoS One. 2014 Jun 18;9(6):e99366. doi: 10.1371/journal.pone.0099366 (PMC4062390; doi:10.1371/journal.pone.0099366)
Supplement: Tables S10 — Prediction of CRE1 binding sites on genes down regulated in a Δcre1 mutant induced with sophorose. (PDF) [file pone.0099366.s010.pdf]

Tables S10. Prediction of CRE1 binding sites on genes down regulated in a *Δcre1* mutant induced with sophorose.

Protein ID	Site 1*	Site 2	Distance	Description	log2 FC (Δcre1/wt)	p-val
**79816**	-1274	-1263	11	unknown protein; secreted	-5.385291084	2.55192E-14
**65817**	-1389	-1383	6	GT α-1,3-mannosyltransferase CMT1	-2.231489889	0.003331794
**45369**	-253	-234	19	unknown protein	-1.992724343	0.027684054
**109394**	-551	-540	11	Zn2Cys6 transcriptional regulator	-1.814824402	4.16711E-05
**105968**	-1071	-1044	27	Iron/ascorbate family oxidoreductases	-1.600400403	1.97426E-05
**105968**	-1044	-1038	6	Iron/ascorbate family oxidoreductases	-1.600400403	1.97426E-05
**74020**	-1033	-1013	20	Orotidine 5'-phosphate decarboxylase	-1.506293889	2.40655E-05
**67902**	-103	-75	28	unknown secreted protein	-1.401912143	0.000136991
**54991**	-893	-884	9	short chain dehydrogenase/reductase	-1.33688536	0.002172725
**111102**	-308	-302	6	Phospholipase C, related to Listeria monocytogenesPlcA (Evalue 1.62e-21), has a homologue in Gibberella zeae	-1.250032843	0.004555341
**111102**	-302	-296	6	Phospholipase C, related to Listeria monocytogenesPlcA (Evalue 1.62e-21), has a homologue in Gibberella zeae	-1.250032843	0.004555341
**65965**	-350	-326	24	unknown protein	-1.245144034	0.020763966
**65209**	-583	-576	7	unknown protein	-1.215083263	0.003346476
**64330**	-1360	-1346	14	indoleamine 2,3-dioxygenase	-1.123158182	0.017803494
**121091**	-1179	-1154	25	unknown protein	-1.089910257	0.04052763
**46613**	-161	-155	6	unknown protein	-1.08906378	0.019306546
**80234**	-1405	-1388	17	unknown protein	-1.038954894	0.010277693
**122576**	-1287	-1281	6	unknown protein	-1.025321386	0.006051123
**63558**	-136	-125	11	unknown protein	-1.024669032	0.006300146

*Positions of Sites 1 and 2 are relative to the gene start codon.
