# Supplementary material for: Deciphering the Cis-Regulatory Elements for XYR1 and CRE1 Regulators in Trichoderma reesei
Source: PLoS One. 2014 Jun 18;9(6):e99366. doi: 10.1371/journal.pone.0099366 (PMC4062390; doi:10.1371/journal.pone.0099366)
Supplement: Tables S11 — Prediction of CRE1 binding sites on genes up regulated in a Δcre1 mutant induced with sophorose. (PDF) [file pone.0099366.s011.pdf]

Tables S11. Prediction of CRE1 binding sites on genes up regulated in a *Δcre1* mutant induced with sophorose.

Protein ID	Site 1*	Site 2	Distance	Description	log2 FC (Δcre1/wt)	p-val
**47710**	-802	-793	9	MFS permease	5.469366622	4.09589E-19
**122208**	-563	-545	18	Zn2Cys6 transcriptional regulator	5.191488284	4.56595E-43
**50618**	-1150	-1139	11	MFS permease	3.902106195	0.00012398
**30465**	-158	-152	6	unknown protein, WD-repeats	3.725897747	1.02183E-23
**67035**	-958	-950	8	unknown protein	3.33305303	1.7282E-18
**102437**	-1321	-1312	9	unique protein, secreted	3.28066688	7.45471E-16
**58282**	-1373	-1364	9	esterase family 9	2.874624778	1.93535E-05
**45624**	-750	-742	8	unknown protein	2.568530973	1.56194E-11
**104081**	-657	-651	6	coenzyme F420-dependent N5,N10-methylene tetrahydromethanopterin reductase	2.528127187	0.010505112
**122240**	-337	-328	9	Carnitine o-acyltransferase	2.477747967	1.5277E-05
**47930**	-619	-613	6	Mitochondrial oxoglutarate/malate carrier proteins	2.206416088	0.030496393
**121298**	-1175	-1145	30	formate dehydrogenase	2.062016661	4.69598E-06
**52438**	-1459	-1445	14	unknown protein	2.043845388	0.010394571
**41035**	-1398	-1389	9	G-protein coupled receptor protein, contains Molluscan rhodopsine C-terminal tail, possibly involved in signal transduction	1.983074388	0.015155077
**21873**	-889	-883	6	dihydrounknown protein-acid dehydratase	1.927417882	1.11511E-05
**79345**	-1160	-1151	9	unknown protein, Mpv17/PMP22 family	1.854222421	0.011982586
**68122**	-772	-755	17	MFS permease	1.782944386	0.029145175
**119823**	-678	-669	9	SAM-dependent methyltransferases	1.768952938	0.015155077
**122376**	-1095	-1086	9	unknown protein	1.641915423	1.5277E-05
**122886**	-1345	-1332	13	Tubulin beta chain 2	1.573645136	3.08517E-05
**122886**	-1332	-1305	27	Tubulin beta chain 2	1.573645136	3.08517E-05
**120826**	-1135	-1119	16	unknown protein	1.568740312	0.00228747
**120826**	-1119	-1094	25	unknown protein	1.568740312	0.00228747

**107187**	-1093	-1077	16	unknown protein	1.557728986	0.000267422
**123227**	-1379	-1369	10	unknown protein	1.517397555	0.002920578
**45912**	-1240	-1228	12	60s ribosomal protein L36 based on homology to the corresponding protein in other fungi.	1.511037344	0.000532228
**76136**	-166	-140	26	unknown protein	1.502298385	0.003329457
**81676**	-463	-457	6	salicylate hydroxylase	1.40398715	0.000248049
**34112**	-1179	-1166	13	ribosomal protein L22.	1.379697699	2.40481E-05
**121074**	-194	-188	6	homeobox transcriptional regulator	1.357140841	0.014697572
**47221**	-30	-21	9	Nucleoside diphosphate kinase	1.319151266	0.000188665
**123771**	-48	-36	12	GCD, Glutaryl-CoA dehydrogenase	1.245877242	0.001931575
**75890**	-1466	-1441	25	C1 tetrahydrofolate synthase, putative	1.160734188	0.000773167
**79329**	-306	-276	30	MFS permease	1.15937328	0.006300146
**79329**	-276	-264	12	MFS permease	1.15937328	0.006300146
**80231**	-545	-528	17	glucokinase	1.146707514	0.006227884
**80231**	-133	-127	6	glucokinase	1.146707514	0.006227884
**121915**	-894	-884	10	Translationally-controlled tumor protein homolog	1.126868825	0.001284327
**121820**	-279	-270	9	Methionine_syntMethionine synthasevitamin-B independent	1.105413674	0.002007961

*Positions of Sites 1 and 2 are relative to the gene start codon.

References

1. Castro, L.D., Antonieto, A.C., Pedersoli, W.R., Rocha, R.S., Persinoti, G.F. and Silva, R.N. (2014) Expression pattern of cellulolytic and xylanolytic genes regulated by transcriptional factors XYR1 and CRE1 are affected by carbon source in Trichoderma reesei. *Gene expression patterns : GEP*.
